# Novel LncRNA LINC02936 Suppresses Ferroptosis and Promotes Tumor Progression by Interacting with SIX1/CP Axis in Endometrial Cancer

**DOI:** 10.7150/ijbs.86256

**Published:** 2024-01-27

**Authors:** Zihui Zhang, Bingshu Li, Zhi Wang, Lian Yang, Jiaxin Peng, Haoyu Wang, Ying Wang, Li Hong

**Affiliations:** Department of Gynecology and Obstetrics, Renmin Hospital of Wuhan University, Wuhan, Hubei Province People's Republic of China.

**Keywords:** lncRNA, LINC02936, SIX1, Ceruloplasmin, Ferroptosis, Endometrial cancer

## Abstract

Endometrial cancer (EC) is a prevalent gynecological malignancy, and metabolic disorders are among its most significant risk factors. Abnormal iron metabolism is associated with the progression of cancer malignancy. Nevertheless, the involvement of iron metabolism in the EC remains uncertain. Ceruloplasmin (CP) functions as a multicopper oxidase and ferroxidase, playing a crucial role in maintaining the metabolic balance between copper and iron. Prior research has demonstrated that the dysregulated expression of CP has important clinical implications in EC. However, ​the specific underlying molecular mechanisms remains uncertain. This research examined the impact of CP on the malignant advancement of EC by suppressing ferroptosis. Next, we explored the possibility that Long non-coding RNA (lncRNA) LINC02936/SIX1/CP axis may be a key pathway for inhibiting ferroptosis and promoting cancer progression in EC. Mechanistically, SIX1 modulates the expression of CP, whereas LINC02936 interacts with SIX1 and recruits SIX1 to the CP promoter, leading to upregulation of CP, inhibition of ferroptosis, and promotion of EC progression. Administration of a small peptide cloud block the LINC02936‐SIX1 interaction, thereby inhibits EC progression by promoting ferroptosis. Altogether, this is the first report on the lncRNA regulation of ferroptosis in EC. Our research enhances the knowledge of the lncRNA-mediated regulation of ferroptosis in EC progression and indicates the potential therapeutic significance of the LINC02936/SIX1/CP axis in treating EC.

## Introduction

Endometrial cancer (EC) is a common gynecological malignancy that exhibits an increasing global incidence 1. Advanced age, obesity, menstrual shampooing, polycystic ovary syndrome, estrogen therapy without progesterone antagonists or tamoxifen, and Lynch syndrome were identified as risk factors for EC 2. Studies has demonstrated a positive correlation between body mass index and the incidence of EC 3. Additionally, polycystic ovary syndrome is greatly related with the development of EC 4. The findings revealed that metabolic disorders play a critical function in the onset and progression of EC.

Iron metabolism serves a particular responsibility in multiple physiological and pathophysiological processes, including oxygen transport and oxidative stress 5. Intracellular iron balance is regulated by various mechanisms including intracellular iron uptake, storage, and excretion 6. However, studies have revealed that abnormal iron metabolism is also associated with tumorigenic processes. For example, the transferrin receptor (TFRC), a crucial transport protein in iron metabolism, exhibits abnormal expression in numerous tumors compared to normal tissues 7, 8. More importantly, TFRC levels positively correlate with the grade, stage, and prognosis of various tumors, including breast cancer 9, glioma 10 and lung adenocarcinoma 11. Hence, targeting iron metabolic pathways could represent a potential therapeutic strategy for tumors. Ferroptosis is a newly recognized form of programmed cell death, primarily characterized by the accumulation of lipid peroxides dependent on iron, which is mainly caused by iron overload and excessive reactive oxygen species (ROS) production 12. MGST1, a ferroptosis-associated MGST1 gene, is highly expressed in endometrial carcinoma, resulting in poor outcome 13. Moreover, the transcription factor ELK1 suppresses ferroptosis and advances the malignant progression of EC through the upregulation of GPX4 expression 14. Therefore, clarifying the key mechanisms that regulate iron metabolism may provide a strategy for EC therapy.

Ceruloplasmin (CP) is widely considered an effective protective agent against antioxidant stress. The mechanism by which CP protects against oxidative stress involves the suppression of Fe2⁺-mediated ROS production of reactive oxygen species. Inhibition of CP expression promotes ferroptosis and radiosensitivity in liver cancer cells 15, 16. Additionally, elevated CP levels are closely associated with poor prognosis in various cancers 17, 18. In addition, serum CP concentration is markedly increased in EC and correlates positively with the advanced stage 19. However, the precise molecular mechanisms by which CP regulate EC malignant progression remains unclear. Thus, we explored that long non-coding RNA (lncRNA) LINC02936/SIX1/CP axis is a key pathway for suppressing ferroptosis and promoting cancer progression in EC. Mechanistically, LINC02936 enhances CP expression by binding to the transcription factor SIX1, which reduces intracellular Fe2+ and ROS levels and suppresses ferroptosis in EC, thus promoting tumor progression. Taken together, we uncovered a new role for the LINC02936/SIX1/CP axis and presented an appealing therapeutic target for EC patients.

## Methods

### Data collection and processing

Gene expression and related clinical data for EC were extracted from The Cancer Genome Atlas-Uterine Corpus Endometrial Carcinoma (TCGA-UCEC) (https://portal.gdc.cancer.gov). Differentially expressed genes were identified through the use of the “edgeR” package between different groups. The diagnostic and prognostic ROC analyses were conducted using the “pROC” and “timeROC” packages, respectively. Pearson correlation was analyzed between CP and lncRNAs and transcription factors. In addition, 238, 264, and 9 ferroptosis suppressor, driver, and marker genes, respectively, were obtained from the FerrDb database 20.

### Cell culture and reagents

The Ishikawa, RL-952, HEC1A, and HEC1B cell lines, along with the endometrial stromal cells (ESCs), were obtained from the China Center for Type Culture Collection (Hubei, China). The KLE cell line was obtained from the Laboratory of Obstetrics and Gynecology, Tongji Hospital, Tongji Medical College, Huazhong University of Science and Technology. Under conditions of 5% CO2 and 37°C, Ishikawa, RL-952, HEC1A, and HEC1B, as well as the ESC cells, were cultured in DMEM medium, and the KLE cells were cultured in DMEM-F12 medium supplemented with 1% penicillin-streptomycin and 10% fetal bovine serum, respectively. In addition, erastin (HY-15763) and ferrostatin-1 (HY-100579) were obtained from MedChem Express.

### CCK-8 assay

Cells were introduced into 96-well dishes and permitted to proliferate for the specified durations. Each well added 10 μl of CCK-8 solution (40203ES76, Yeasen) and incubated for a duration of 2h. The cell viability was assessed by quantifying the optical density (OD) of each well at a wavelength of 450 nm using a microplate reader (EnSight, USA).

### Plate cloning experiment and transwell invasion assay

The cell proliferation as well as invasion ability were evaluated by the plate cloning and transwell invasion assays 21. ImageJ software (v.1.8.0) was used to quantify the results.

### Malondialdehyde (MDA) assay

A Lipid Peroxidation Assay Kit (S0131S, Beyotime) was utilized to assess the relative MDA levels, according to the manufacturer's instructions.

### Lipid ROS assay

Cells from each experimental group were seeded into 6-well plates. Serum-free medium containing BODIPY 581/591 C11 probe (RM02821, ABclonal) was added and incubated for 1 h away from light. Subsequently, cells were digested using pancreatic enzymes and analyzed by flow cytometry (CytoFlex, Beckman Coulter) following the instructions provided by the manufacturer.

### Ferrous iron assay

Ferrous iron ion probe (FerroOrange, F374, Dojindo) was utilized to measure the intracellular relative Fe^2+^ levels. Following the protocol, the cells were treated with FerroOrange at a working concentration of 1 μmol/L for 0.5 h. Fluorescence intensity was assessed by a fluorescence microplate reader (EnSight, Perkin Elmer).

### Gene over-expression and knockdown

Ribobio (Guangzhou, China) provided the overexpression vectors for Human *LINC02936* (ENSG00000227359), *SIX1* (ENSG00000126778), and *CP* (ENSG00000047457), as well as an empty vector containing pGPU6. *LINC02936* (sh-*LINC02936* #1 and sh-*LINC02936* #2), *SIX1* (sh-*SIX1* #1 and sh-*SIX1* #1), and *CP* (sh-*CP* #1 and sh-*CP* #2) were purchased from RiboBio (Guangzhou, China) with pGPU6 vector. The target sequences of shRNA used in this study were *LINC02936* shRNA#1: AGGGCCGTAAACCAGTTATTA; shRNA#2: GACCAAGAACTTCGCTTTATT; *SIX1* shRNA#1: AGCTTGTTTCTGGAGTTGTTT; *SIX* shRNA#2: CCAGACCAGAACTCGGTCCTT; *CP* shRNA#1: GGAATTATTGAAACGACTTGG; *CP* shRNA#2: GGAGCTGCATCATTTACAAGA.

### Real-time quantitative RT-PCR (RT-qPCR)

RT-qPCR was executed as previously reported 21. The primer sequences utilized for RT-qPCR were displayed in **Table S1**.

### Western blotting assay

In short, western blotting assay was carried out as described earlier 21, with rabbit anti-human antibodies for SIX1 (1:1000, 10709-1-AP, Proteintech), ZNF516 (1:1000, A303-392A, Bethyl), GLIS2 (1:1000, PA5-40314, Thermo Fisher), CEBPG (1:500, 12997-1-AP, Proteintech), MESP2 (1:1000, PA5-75751, Thermo Fisher), IKZF3 (1:500, A8614, ABclonal), SIX3 (1:1000, A17363, ABclonal), MEOX1(1:1000, A7332, ABclonal), CP (1:1000; 21131-1-AP, Proteintech), β-actin (1:2000, GB11001, Servicebio), Flg-tag (2μg/ml, PK10315, Abmart) or mouse anti-human antibodies for GST-tag (1:3000, MA9024, Abmart). After incubation, the membrane was incubated with goat anti-rabbit (1:3000, SA00001-2, Proteintech) or goat anti-mouse (1:4000, SA00001-1, Proteintech) at room temperature for 2 h and immediately visualized using a chemiluminescence imaging system.

### RNA-fluorescence *in situ* hybridization (FISH)

A FISH kit (R0306S, Beyotime) was employed in accordance with the manufacturer's instructions. KLE cells were immobilized by 4% formaldehyde for 10 minutes and 0.5 μg/ml LINC02936 FISH probe was hybridized at 37 °C overnight. Then, the nucleus was stained with DAPI, and photographs were taken using a confocal microscope (FV1200, Olympus).

### RNA-pull down

A commercial kit (20164, Pierce USA) was used for transcriptional synthesis of biotin-labeled RNAs *in vitro*. Biotin-labeled RNA (20 pmol) and streptavidin magnetic beads (30 µL) were then incubated with nuclear extracts, following the manufacturer's instructions. A magnetic stand was introduced to collect the precipitated components, and sodium dodecyl sulfate-polyacrylamide gel electrophoresis and western blotting were used to separate the proteins.

### RIP assays

Anti-SIX1 antibody (1:500, 10709-1-AP, Proteintech) and purified rabbit IgG (1:500, 30,000-0-AP, Proteintech) were used for the RIP assay. In brief, 1 × 10^7^ cells were cleaved in 0.1 mL of RIP lysis buffer containing protease inhibitors and RNase inhibitors and centrifuged at 12000 rpm on 4 °C for 15 min. The supernatant and magnetic beads were incubated with 3 μg anti-SIX1 or rabbit IgG negative control at 4 °C for 12h. Then, the beads were washed with cold RIP wash buffer, and the purified RNA was used for quantitative real-time PCR.

### RNA electrophoretic mobility shift assay (EMSA) assays

SIX1 human fusion protein GST was purchased from Proteintech (Ag1044). Biotin-labeled RNA probes for LINC02936 exons were synthesized *in vitro* following the manufacturer's instruction (20164, Pierce USA). The probe was then incubated with the SIX1-GST protein. Finally, RNS-EMSA was performed according to the manufacturer's instructions (20158, Thermo Fisher Scientific).

### Luciferase reporter assay

In brief, the human CP promoter (-2000/+99) was amplified from genomic DNA using PCR, and subsequently inserted into the pGL3-Basic vector (Promega, USA). Measure the luciferase activity as described earlier 21. The primer sets for the *CP* promoter (-1761/-1586) were listed as follows: 5'- TCCTAGAAGGAGGCCTTGGG -3' (forward) and 5'- TACTTCAGCCCAGCTTCAGTTTCC-3' (reverse).

### ChIP assay

ChIP-qPCR was carried out by the EZ-CHIP Kit (Millipore, USA) following the previously described method 21. The primer sequences were provided in **Table S1**.

### Transmission electron microscopy

Cells in each group were fixed with 1 ml fixative solution ultrathin sections (65 nm) of fixative solution and stained with 1% uranyl acetate and 0.1% lead citrate. Morphological changes in the mitochondria were captured using transmission electron microscopy.

### Hematoxylin and eosin (HE) staining and immunohistochemistry (IHC)

Briefly, HE and IHC protocols were carried out as previously reported 22. The sections were incubated with rabbit anti-human SIX1 (1:100, 10709-1-AP, Proteintech), CP (1:100, 21131-1-AP, Proteintech), Ki-67 (1:10000, 27309-1-AP, Proteintech), subsequent incubation with a biotinylated goat anti-rabbit antibody. Then, three random different fields (X200) were captured using OLYMPUS (BX53). SIX1 and CP were quantified based on the percentage (0: 0 - 24%; 1: 25 - 49%; 2: 50 - 74%; 3: 75 - 100%) and intensity (0: absent; 1: weak; 2: 50 - 74%; strong; 3: very strong) of positive cells. IHC score = percentage score + intensity score. Ki-67 was quantified by calculating the percentage of positive cells.

### * In vivo* experiment

The *in vivo* experiment schemes were authorized by the Animal Research Ethics Committee of the Renmin Hospital of Wuhan University. 1 × 10^6^ tumor cells were injected into the right dorsal flank of female BALB/c nude mice, with 5 mice per group aged 4 weeks. Tumor volume was evaluated every seven days using a caliper and calculated as length × (width)^2^/2.

Furthermore, a total of 1 × 10^7^ cells were injected into the caudal vein of each mouse, with 5 mice per group in the *in vivo* lung metastasis experiment. Immediately, we randomly counted the number of lung metastatic nodules after HE staining.

### EC and normal endometrium tissues

Forty-eight EC patients and matched normal endometrial tissues were obtained from the Department of Gynecology and Obstetrics, Renmin Hospital of Wuhan University. None of the patients had received specific treatment, such as chemotherapy or radiotherapy, prior to the surgery. Meanwhile, informed consent was obtained from each patient. This research was authorized by the Ethics Committee of the Renmin Hospital of Wuhan University. The procedures for HE staining, IHC, and WB have been described previously.

### Statistical analysis

Measurement data are presented as mean ± standard deviation. A two-tailed unpaired t-test was used to determine the statistical significance when comparing two independent groups. ​The paired groups were analyzed using paired t-test. Post-hoc Bonferroni's test after one-way ANOVA analysis was utilized for comparing significance across multiple comparisons. Count data were expressed as the number of cases and performed using a χ^2^ test or Fisher's exact test. For survival significance analysis, we split the patients using the median as a cut-off value and compared them using the log-rank test. Pearson correlation and all statistical analyses were performed using GraphPad 9.0 (GraphPad, Inc., USA), and statistical significance was considered when *P* < 0.05.

## Results

### CP exhibits high expression level in EC and contributes to cancer progression

**Figure 1:**
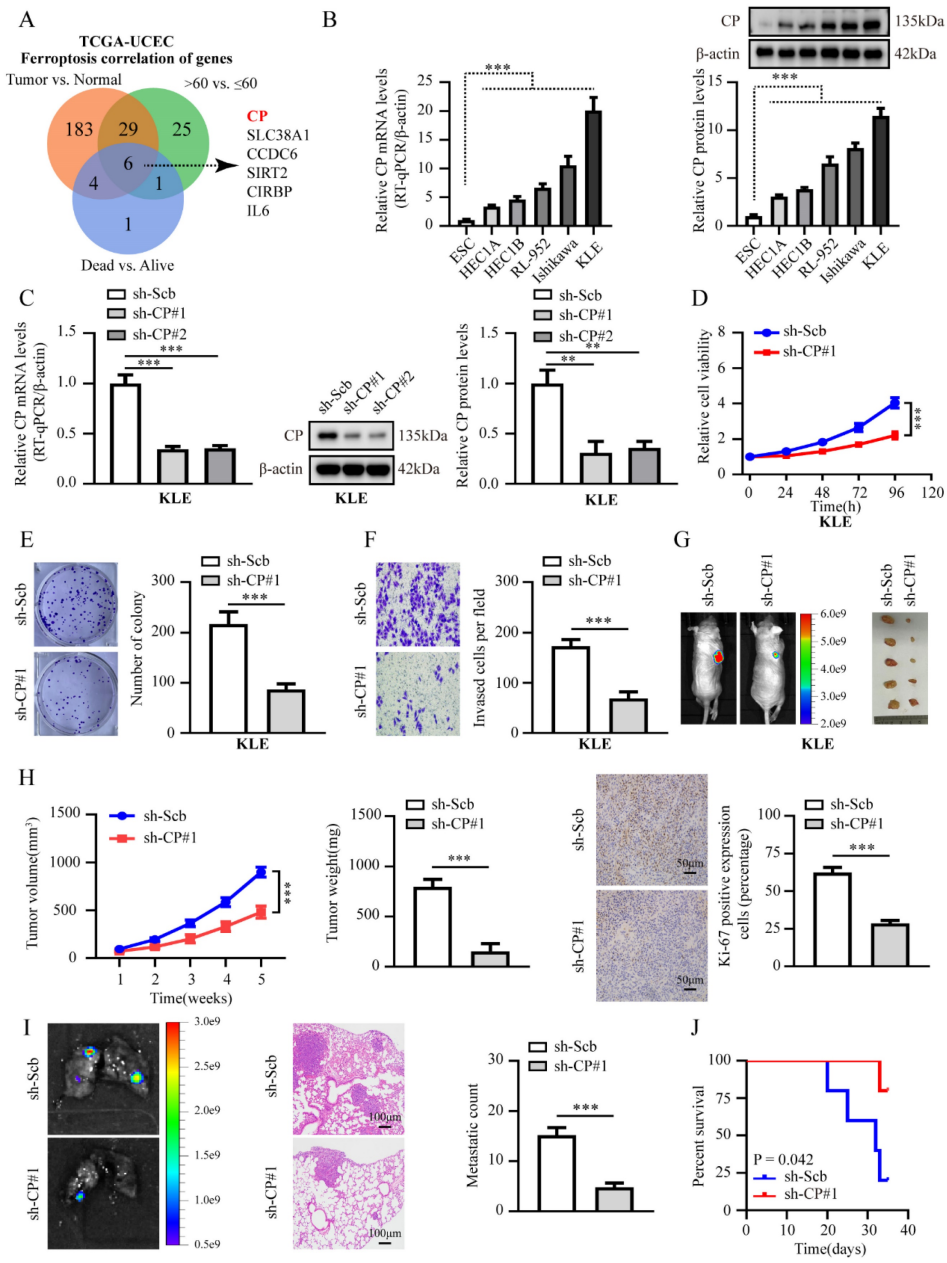
**CP is markedly expressed in EC and promotes tumor progression.** (**A**) Venn diagram indicating ferroptosis genes differentially expressed in TCGA-UCEC database between tumor vs. normal, >60 years old vs. ≤ 60 years old, and dead vs. alive, respectively (FDR < 0.01). (**B**) Relative mRNA and protein levels of CP between EC cell lines and ESC line were detected by RT-qPCR (left panel) and western blot (right panel), respectively. (**C**) RT-qPCR was applied to detect the relative expression of CP in KLE cells stably transfected with sh‐Scb, sh‐CP #1 and sh‐CP #2 (n = 3) (left panel) and western blot (n = 3) (right panel), respectively. (**D-F**) CCK-8 (**D**), plate cloning (**E**) and matrigel invasion (**F**) assays assess the relative cell ability, proliferation and invasion capacity in KLE cell lines stably transfected with sh‐Scb, sh‐CP#1 (n = 3), respectively. (**G-H**) Representative images of xenograft tumors (**G**), tumor growth (left panel), weight (middle panel), Ki-67 percentage (right panel, scale bar: 50 μm) in nude mice injected KLE cells stably transfected with sh‐Scb and sh‐CP#1 (n = 5 per group), respectively. (**I**) Representative images of lungs (left panel, scale bar: 100 μm), metastatic counts (right panel) in nude mice injected KLE cells into the caudal vein with stably transfected with sh‐Scb and sh‐CP#1 (n = 5 per group), respectively. (**J**) The Kaplan-Meier curves illustrate the survival outcomes of nude mice in an experimental metastasis assay (n = 5 per group). Post-hoc Bonferroni's test after one-way ANOVA analysis was applied in panel **B** and **C**. Student's t test compared the difference in panel **E**, **F**, **H** and **I**. Log-rank test for the survival analysis in panel **J**. * *P* < 0.05; ** *P* < 0.01; *** *P* < 0.001.

Research has indicated that older age is a crucial risk factor for EC patients, with the mean age at the time of diagnosis being 60 years 23. Furthermore, ferroptosis-associated gene signature can efficiently estimate the prognosis of patients with EC 24. Thus, it is imperative to investigate the significant impact of ferroptosis genes in EC, which could hold diagnostic and prognostic value and contribute to tumor progression. Therefore, we performed comprehensive analysis and identified 222, 61, and 12 differentially expressed ferroptosis genes (FDR < 0.01) in TCGA-UCEC with tumor vs. normal, >60 years old vs. ≤ 60 years old and dead vs. alive, respectively. Based on the overlap of these results, six ferroptosis genes were consistently related to the tumor, age, and OS events of EC (**Fig. 1A**). Due to the highest fold change between these groups, CP caught our particular attention, and further analysis indicated consistent high expression of CP in tumor tissues, > 60 years old, G3, and the dead group (**Table S2**) (**Fig. S1A**). More importantly, extreme CP manifestations were positively correlated with poor survival (**Fig. S1A**). Then, to further determine the expression pattern of CP in EC, we explored CP expression based on GENT2 25, and found that CP was consistently overexpressed in EC than that in normal tissues (**Fig. S1B**). Furthermore, we explored the diagnostic and prognostic significance of CP expression in EC and showed that the AUC values based on TCGA-UCEC were 0.812 and 0.724, respectively (**Fig. S1C-D**). Those results suggested that CP could be a diagnostic factor and efficiently estimate the prognosis for EC patients.

Next, we examined CP expression between five EC cell lines and ESC line, and discovered CP was highly expressed in EC cell lines (**Fig. 1B**). To further characterize the effect of CP in EC, we chose KLE and HEC1A cells to establish stable knockdown and overexpression of CP, respectively (**Fig. 1C, Fig. S2A**). As illustrated in **Fig. 1D-F** and **Fig. S2B-D**, silencing or over-expression of CP resulted in reduced or enhanced cell viability, proliferation, and invasion capacity in KLE and HEC1A cells, respectively.

To determine the carcinogenesis of CP in EC *in vivo*, we established a xenograft tumor and lung metastasis assay in nude mice. ​For the results shown in **Fig. 1G-J and Fig. S2E-H**, knockdown or ectopic overexpression of CP significantly reduced or increased tumor growth, weight, Ki-67 percentage, lung metastatic nodules count, and extended or shorten survival time, respectively. In short, those data indicated the oncogenic role of CP in EC progression.

### Knockdown of CP inhibits the malignancy progression in EC by promoting ferroptosis

**Figure 2:**
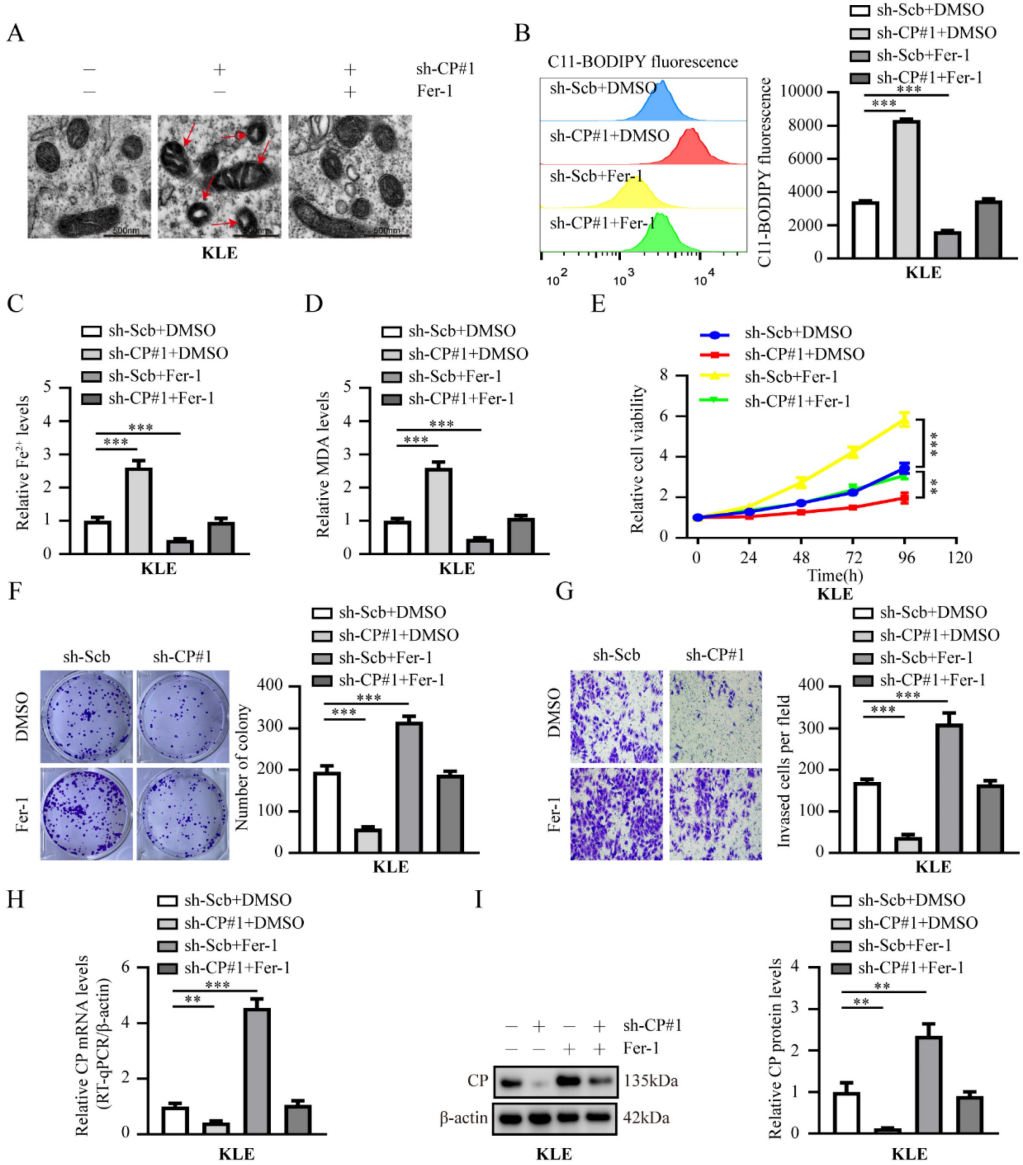
** Knockdown of CP inhibits EC tumor progression by promoting ferroptosis in KLE cells.** (**A**) The distinctive changes in mitochondrial morphology in KLE cell lines after treatment with sh-Scb combine with DMSO, sh-CP#1 combine with DMSO or sh-CP#1 combine with ferrostatin-1 (Fer-1, 20 μM), respectively. Scale bar, 500 nm. (The red arrow represents the ferroptosis characteristic mitochondria). (**B-D**) Relative lipid ROS (**B**), Fe^2+^ (**C**) and MDA (**D**) levels were evaluated in KLE cells stably transfected with sh‐Scb, sh‐CP#1 or co-treatment with DMSO, ferrostatin-1 (Fer-1, 20 μM), respectively (n = 3). (**E-G**) The cell ability (**E**), proliferation (**F**) and invasion ability (**G**) were assessed in KLE cell lines transfected with sh‐Scb, sh‐CP#1 and those co-treatment with DMSO, ferrostatin-1 (Fer-1, 20 μM), respectively (n = 3). (**H-I**) RT-qPCR (**H**) and western blot (**I**) revealed the mRNA and protein levels of CP in KLE cells transfected with sh-Scb, sh-CP#1 and co-treatment with DMSO, ferrostatin-1 (Fer-1, 20 μM), respectively (n = 3). Post-hoc Bonferroni's test after one-way ANOVA analysis was applied in panel** B-I**. * *P* < 0.05; ** *P* < 0.01; *** *P* < 0.001.

Given that CP expression is closely related to ferroptosis 15, 16, and that CP is a ferroptosis suppressor gene based on the FerrDb database 20, we speculated that the elevated expression of CP might be related to the suppression of ferroptosis and promotion of tumor progression in EC cells. As expected, distinctive changes in the mitochondrial morphology of ferroptosis were observed upon inhibiting CP expression in KLE cells. However, ferrostatin-1 (Fer-1, a ferroptosis inhibitor) rescued the changes in mitochondrial morphology generated by silencing CP (**Fig. 2A**). In addition, CP overexpression abolished the changes in mitochondrial morphology induced by erastin (a ferroptosis activator) (**Fig. S3A**). Subsequently, we investigated indicators of ferroptosis in each group, including lipid ROS, Fe^2+^, and MDA. The results revealed that silencing CP notably increased lipid ROS, Fe^2+^, and MDA levels in KLE cells, and these changes could be reversed by Fer-1 (**Fig. 2B-D**). In contrast, CP overexpression significantly reduced lipid ROS, Fe^2+^, and MDA levels in HEC1A cells, and these effects could be reversed by erastin (**Fig. S3B-D**). More importantly, Fer-1 reversed EC cell viability, proliferation, and invasion induced by CP knockdown in KLE cells (**Fig. 2E-G**). Contrarily, erastin inhibited EC cell viability, proliferation, and invasion, which were increased by CP overexpression in HEC1A cells (**Fig. S3E-G**). Additionally, we detected the mRNA and protein levels of CP in each group, and the results suggested that Fer-1 or erastin increased or decreased the expression of CP, respectively (**Fig. 2H-I**, **Fig. S3H-I**), which may be responsible for Fer-1 to reverse ferroptosis or erastin-induced ferroptosis. Previous study has reported that ferroptosis activators (Erastin and RSL3) can promote ferroptosis by inhibiting CP expression in liver cancer 15, our study indicated similar results. Another study revealed that Fer-1 at a certain concentration can promote cell viability 26. However, the phenomenon of increased CP expression by Fer-1 was discovered for the first time and its specific mechanism requires further investigation. In summary, these findings indicated that CP promoted tumor progression by inhibiting ferroptosis in EC.

### LINC02936 inhibits ferroptosis and promotes tumor progression by up-regulating CP expression

**Figure 3:**
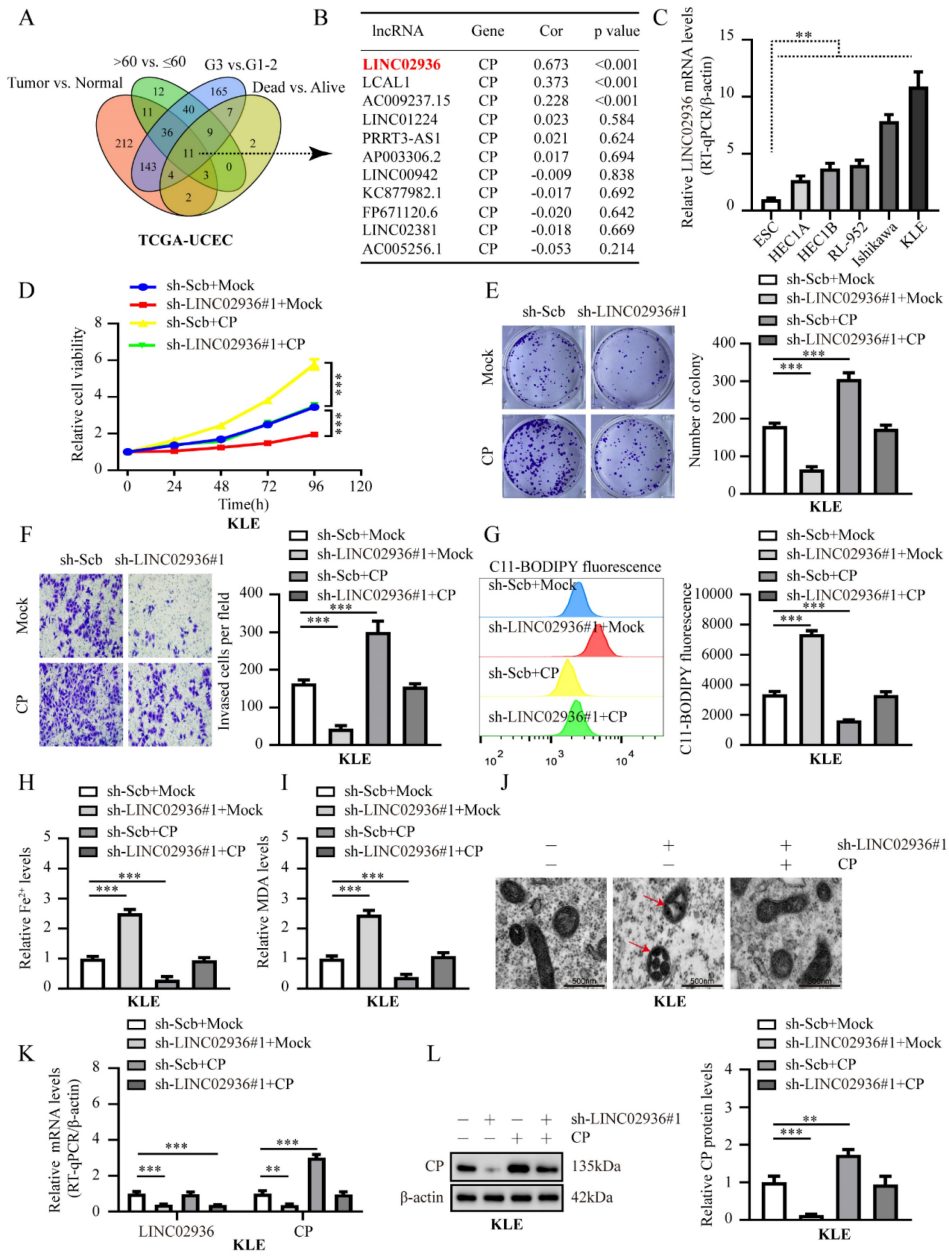
** Knockdown of LINC02936 suppress EC progression by CP-mediated ferroptosis in KLE cells.** (**A**) Venn diagram displayed the differentially expressed lncRNA between tumor vs. normal, >60 years old vs. ≤ 60 years old, G3 vs. G1-2, and dead vs. alive in TCGA-UCEC database, respectively (FDR < 0.01). (**B**) Pearson correlation analysis between eleven different lncRNAs and CP in TCGA-UCEC database. (**C**) Relative expression of LINC02936 between EC cell lines and ESC were tested by RT-qPCR (n = 3). (**D-F**) The cell ability (**D**), proliferation (**E**) and invasion ability (**F**) in KLE cell lines were evaluated transfected with sh‐Scb, sh‐LINC02936#1 and co‐transfected with mock or CP, respectively (n = 3). (**G-I**) Relative lipid ROS (**G**), Fe^2+^ (**H**) and MDA (**I**) levels were measured in KLE cells stably transfected with sh‐Scb, sh‐LINC02936#1 and those co‐transfected with mock or CP, respectively (n = 3). (**J**) The distinctive changes in mitochondrial morphology in KLE cell lines after treatment with sh-Scb combine with mock, sh‐LINC02936#1 combine with mcok or sh‐LINC02936#1 combine with CP, respectively. Scale bar, 500 nm. (The red arrow represents the ferroptosis characteristic mitochondria). (**K**) Relative mRNA expression of LINC02936 and CP in KLE cell lines stably transfected with sh‐Scb, sh‐LINC02936#1 and those co‐transfected with mock or CP by RT-qPCR, respectively (n = 3). (**L**) Relative protein expression of CP in KLE cell lines stably transfected with sh‐Scb, sh‐LINC02936#1 and those co‐transfected with mock or CP by western blot (n = 3), respectively. Post-hoc Bonferroni's test after one-way ANOVA analysis was applied in panel **C-I** and **K-L**. * *P* < 0.05; ** *P* < 0.01; *** *P* < 0.001.

Previous literature has clarified the significant role and mechanism of lncRNAs in regulating ferroptosis 27. Regrettably, the precise mechanism by which lncRNAs regulate ferroptosis and tumor progression in EC remains unknown. Recent study has shown that LINC00176 promotes ovarian cancer progression by up-regulating the CP expression 28. Based on the above research background, we comprehensively analyzed the differentially expressed lncRNAs between the different groups (FDR<0.01). As shown in **Fig. 3A-B**, eleven lncRNAs were consistently correlated with tumor, age, grade, and survival status (**Table S3**), and the association between CP and LINC02936 exhibited the highest degree of correlation (*R* = 0.673, *P* < 0.001). Additionally, LINC02936 was consistently extremely expressed in tumor tissues, > 60 years old, G3, dead group, and had poor survival outcome (**Fig. S4A**), which was consistent with CP in TCGA-UCEC. Further analysis revealed a low coding capacity of LINC02936 29. We next explored the expression level of LINC02936 in EC cell lines and ESC by RT-qPCR, and the results suggested that LINC02936 was extremely expressed in EC cells (**Fig. 3C**). Further functional experiments suggested that knockdown of LINC02936 significantly suppressed cell viability, proliferation, invasion ability, and increased lipid ROS, Fe^2+^, and MDA levels in KLE cells (**Fig. 3D-I**). However, excessive expression of LINC02936 enhanced cell viability, proliferation, and invasion and reduced lipid ROS, Fe^2+^, and MDA levels in HEC1A cells (**Fig. S4B-G**). In addition, overexpression or knockdown of CP reversed the changes in cell viability, proliferation, and invasion and eliminated lipid ROS, Fe^2+^, and MDA levels, which resulted in knockdown or overexpression of LINC02936 in KLE and HEC1A cells, respectively (**Fig. 3D-I**, **Fig. S4B-G**). Additional changes in mitochondrial characteristics were confirmed by transmission electron microscopy (**Fig. 3J**, **Fig. S4H**). Accordingly, knockdown or ectopic expression of LINC02936 decreased or increased CP levels in KLE and HEC1A cells, respectively (**Fig. 3K-L**, **Fig. S4I-J**). Collectively, these data suggested that LINC02936 suppresses ferroptosis and facilitates the progression of EC tumors by increasing CP expression.

### LINC02936 physically interacts with SIX1 in EC cells

**Figure 4:**
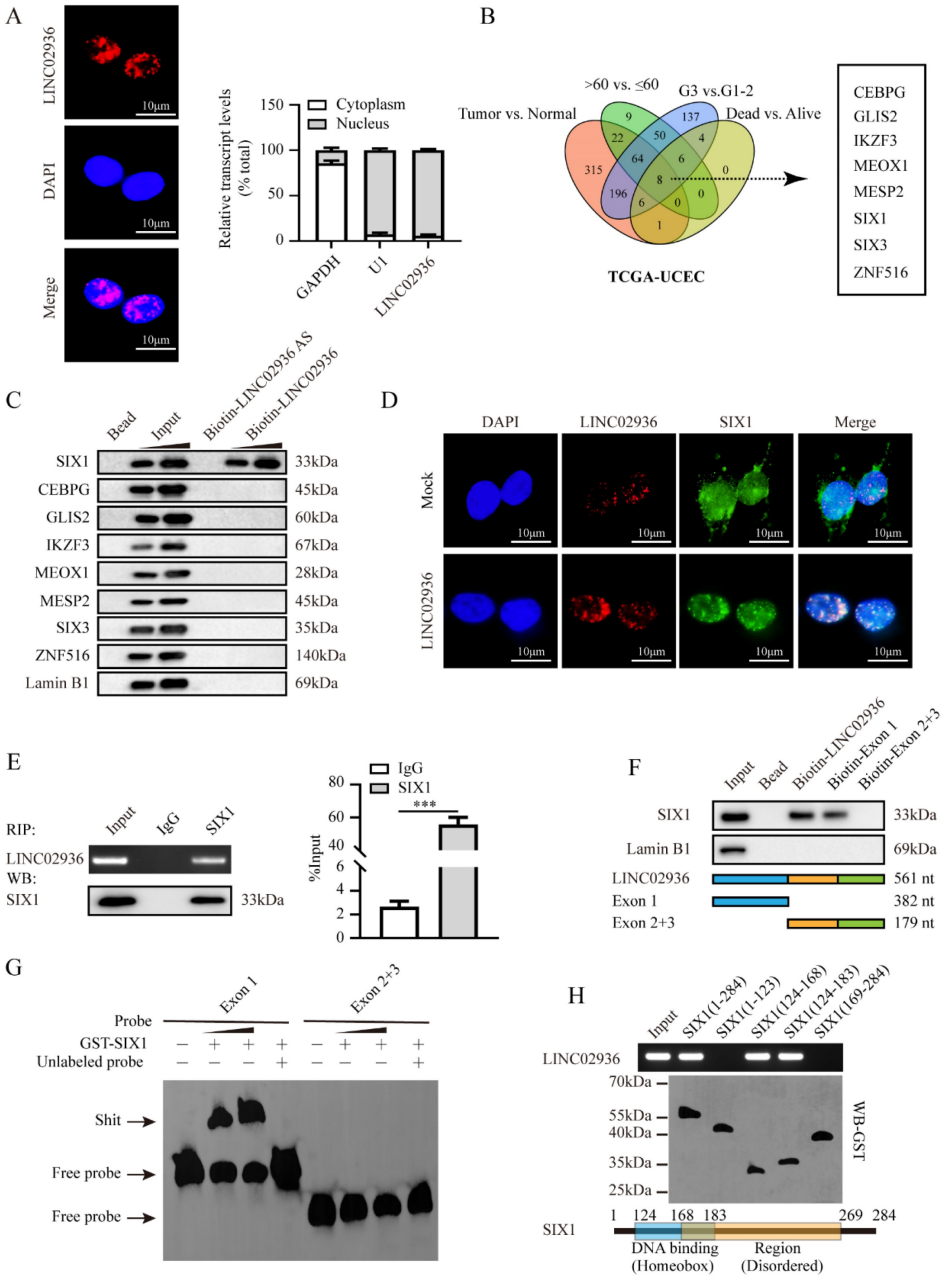
** LINC02936 physically interacts with SIX1 in EC cells.** (**A**) FISH analysis (left panel, red, scale bar: 10 μm) and RT‐qPCR (n = 3) confirmed the localization of LINC02936 within KLE cells (**B**) Venn diagram indicating transcription factors differentially expressed in TCGA-UCEC database with differentially expressed between in tumor vs. normal, >60 years old vs. ≤ 60 years old, G3 vs. G1-2 and dead vs. alive, respectively (FDR < 0.01). (**C**) Western blot after biotin‐labeled RNA pull‐down indicated the protein pulled down by LINC02936 from lysates of KLE cells. The bead-bound protein and LINC02936 antisense (AS) were set as negative controls. (**D**) Confocal images indicated the co‐localization of LINC02936 (red) and SIX1 protein (green) in the nucleus of HEC1A cells. (**E**) Western blot (lower panel) and RIP (upper panel) assays revealed the interaction between LINC02936 and SIX1 protein in KLE cells. (**F**) Biotin‐labeled RNA pull‐down assay (upper panel) indicated the interaction between LINC02936 truncations (lower panel) and SIX1 protein in KLE cells. Bead presented as a negative control. (**G**) EMSA assay revealed biotin‐labeled probes (corresponding to Exon 1) indicating the interaction of LINC02936 with glutathione S‐transferase (GST)‐tagged recombinant SIX1 protein. (**H**) *In vitro* binding assay revealed the LINC02936 levels identified by RT‐PCR (upper panel) following incubation with truncation forms or full‐length of GST‐tagged recombinant SIX1 protein validated by western blot (lower panel). Student's t test compared the difference in panel **E.** * *P* < 0.05; ** *P* < 0.01; *** *P* < 0.001.

To investigate the precise molecular mechanism by which LINC02936 regulates CP expression in EC, we initially determined the cellular localization of LINC02936 by carrying out FISH and nuclear/cytoplasmic RNA fractionation assays. All the data revealed that LINC02936 was primarily localized in the nucleus of KLE cells (**Fig. 4A**). Same result was observed in the RNALocate v2.0 database 30. According to lncRNA localization and function studies 31, LINC02936 may regulate the expression of CP through RNA-binding proteins or transcription factors (TFs) and promote the malignant progression of EC. Previous study has reported that LINC00176 promotes ovarian cancer progression via transcription factor BCL3-mediated upregulation of CP 28. Therefore, we first analyzed the differentially expressed TFs between different EC groups (FDR < 0.01). Finally, a comprehensive analysis identified eight TFs that were consistently associated with tumor, age, grade, and survival status (**Fig. 4B**) (**Table S4**). The RPISeq software 32 was utilized to forecast the interaction of LINC02936 with eight TFs, and the results revealed that LINC02936 may directly interact with them (**Fig. S5A**, **Fig. S5C**). Then, RNA pull-down illustrated that LINC02936 interacts with SIX1 but not with other TFs (**Fig. 4C**). In addition, the colocalization of LINC02936 and SIX1 was observed in HEC1A cells, which was significantly increased by the ectopic expression of LINC02936 (**Fig. 4D**). RIP assay verified the specific binding of SIX1 to LINC02936 in KLE cells (**Fig. 4E**). Further experiments revealed that exon 1 of LINC02936 was responsible for its interaction with SIX1 (**Fig. 4F-G**). The DNA-binding domain, especially the region of amino acids 124-168 amino acids (aa) of the SIX1 protein, was responsible for interacting with LINC02936 (**Fig. 4H**). Those data suggested that LINC02936 physically interacts with SIX1 in EC cells.

Moreover, the expression of SIX1 was positively linked to CP (*R* = 0.154, *P* < 0.001) (**Fig. S5B**). More importantly, the level of SIX1 was consistently high in tumor tissue, > 60 years old, G3, dead group, and poor survival (**Fig. S5D**), which was consistent with CP in the TCGA-UCEC. Thus, our comprehensive analysis suggested that SIX1 may involve in regulating CP expression in EC.

### SIX1 promotes EC progression by CP-mediated iron homeostasis

**Figure 5:**
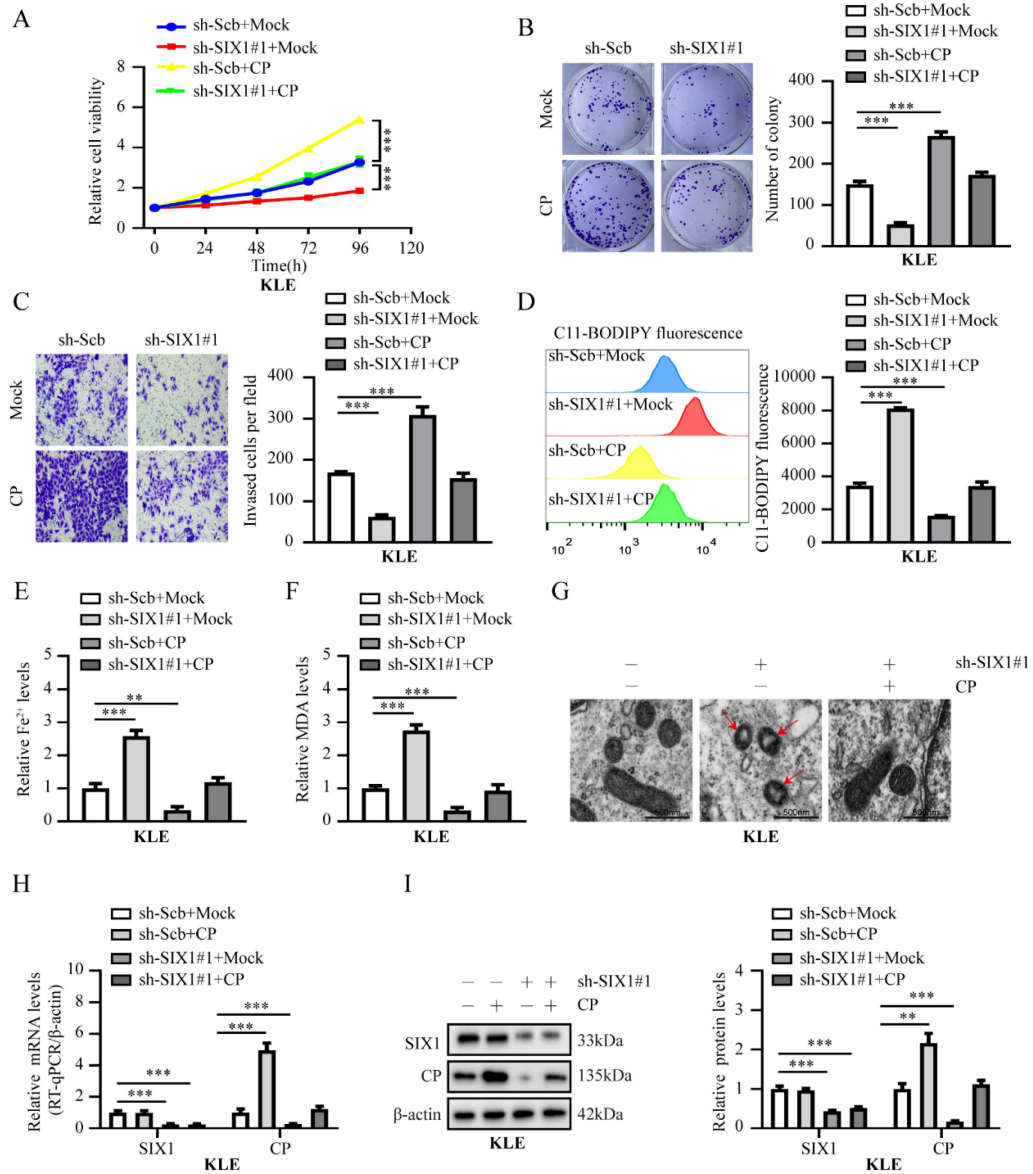
** Knockdown of SIX1 suppress EC progression by CP-mediated ferroptosis in KLE cells.** (**A-C**) The cell ability (**A**), proliferation (**B**) and invasion ability (**C**) in KLE cell lines transfected with sh‐Scb, sh‐SIX1#1 and co‐transfected with mock or CP, respectively (n = 3). (**D-F**) Relative lipid ROS (**D**), Fe^2+^ (**E**) and MDA (**F**) levels were measured in KLE cells transfected with sh‐Scb, sh‐SIX1#1 and co‐transfected with mock or CP, respectively (n = 3). (**G**) The distinctive changes in mitochondrial morphology in KLE cell lines after treatment with sh-Scb combine with mock, sh‐SIX1#1 combine with mcok or sh‐SIX1#1 combine with CP, respectively. Scale bar, 500 nm. (The red arrow represents the ferroptosis characteristic mitochondria). (**H-I**) Relative expression of SIX1 and CP in KLE cell lines stably transfected with sh‐Scb, sh‐SIX1#1 and those co‐transfected with mock or CP by RT-qPCR (**H**) and western blot (**I**), respectively (n = 3). Post-hoc Bonferroni's test after one-way ANOVA analysis was applied in panel **A-F** and **H-I**. * *P* < 0.05; ** *P* < 0.01; *** *P* < 0.001.

To elucidate the potential mechanism of CP regulation by SIX1, we stably silenced or expressed SIX1 in KLE and HEC1A cells. As expected, SIX1 knockdown suppressed cell viability, growth, invasion ability, and increased the relative lipid ROS, Fe^2+^, and MDA levels in KLE cells (**Fig. 5A-F**). In contrast, overexpression of SIX1 increased cell viability, growth, invasion ability, and decreased the relative lipid ROS, Fe^2+^, and MDA levels of HEC1A cells (**Fig. S6A-F**). Moreover, overexpression of CP rescued the decrease in cell viability, growth, and invasion and increased the relative lipid ROS, Fe^2+^, and MDA levels induced by stable silencing of SIX1 in KLE cells (**Fig. 5A-F**). In addition, knockdown of CP abolished the increase in cell viability, growth, and invasion and decreased the relative lipid ROS, Fe^2+^, and MDA levels induced by SIX1 overexpression in HEC1A cells (**Fig. S6A-F**). We also confirmed mitochondrial changes in KLE and HEC1A cells using transmission electron microscopy (**Fig. 5G**, **Fig. S6G**). In addition, stable depletion or increased SIX1 expression led to significantly reduced or elevated CP expression in KLE and HEC1A cells, respectively (**Fig. 5H-I. Fig. S6H-I**). Notably, ectopic or knockdown expression of CP abolished the CP gene expression induced by depletion or increased SIX1 expression in KLE and HEC1A cells, respectively (**Fig. 5H-I. Fig. S6H-I**). These results suggested that SIX1 inhibits ferroptosis and promotes malignant progression by up-regulating CP expression in EC.

**Figure 6:**
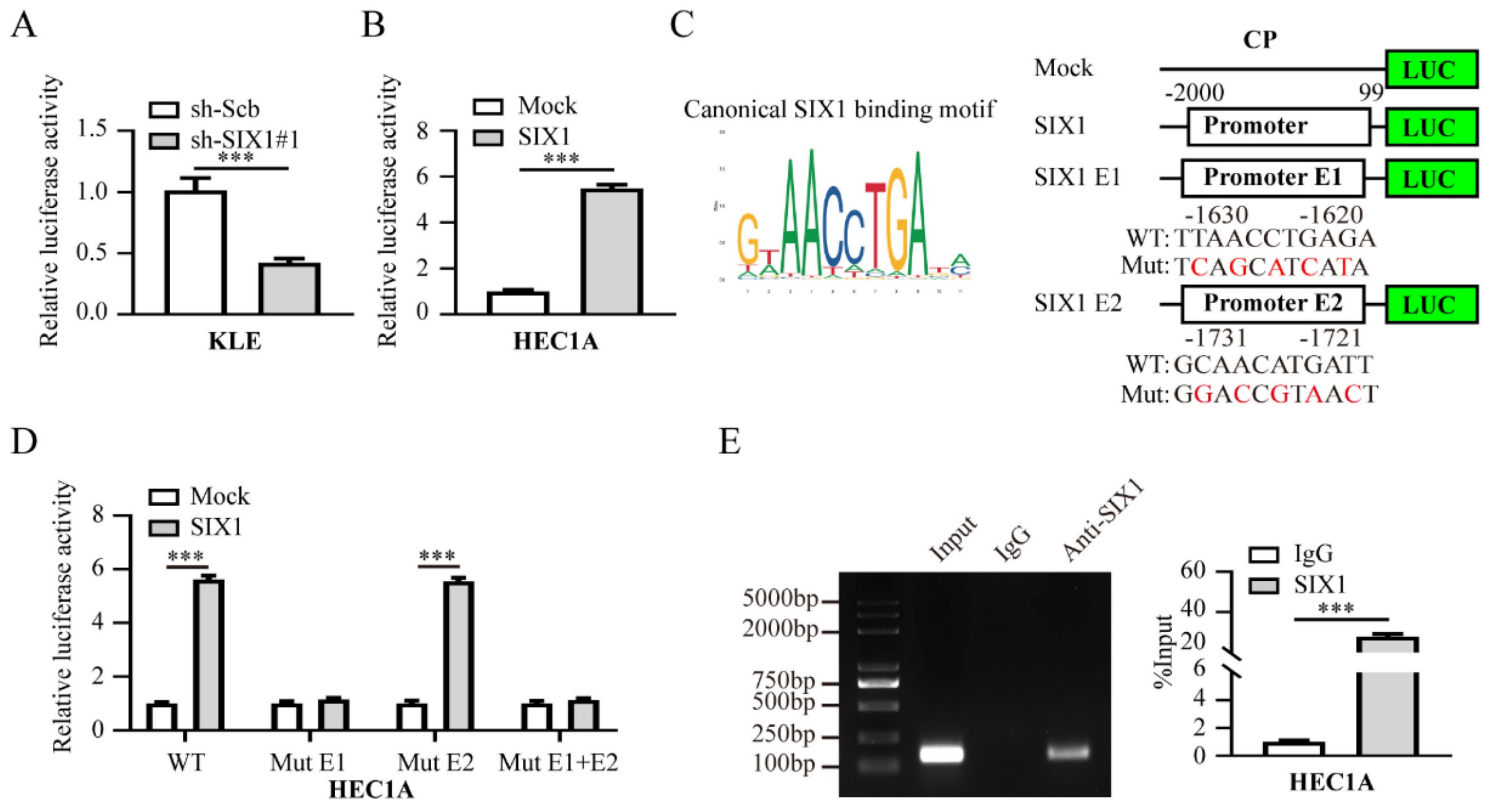
** SIX1 can activate CP transcription in EC cells.** (**A-B**) Luciferase assay was applied to evaluate SIX1 promoter transcriptional activity when cells were transfected with sh-Scb, sh-SIX1#1 in KLE (**A**) or mock, SIX1 in HEC1A (**B**) cells, respectively (n = 3). (**C**) The typical binding motif of SIX1 and two potential SIX1 response elements, E1 and E2, were identified in the CP promoter based on the JASPAR database (https://jaspar.genereg.net/). (**D**) Luciferase assay was applied to evaluate transcriptional activities of the two CP promoter mutations when SIX1 expression was overexpressed in HEC1A cells (n = 3). (**E**) The ChIP assays demonstrated the binding of SIX1 to the CP promoter (n = 3). IgG was set as a negative control for the experiment. The left panel was the electrophoresis result of the ChIP products and the right panel was the result of statistical analysis. Student's t test applied the statistical difference in panel **A**-**B** and **D**-**E**. * *P* < 0.05; ** *P* < 0.01; *** *P* < 0.001.

In order to characterize the molecular mechanism by which SIX1 regulates CP, its effect on the transcriptional activity of CP promoter was examined. As displayed in **Fig. 6A-B**, our results suggested that the promoter activity of CP was increased and attenuated by altered levels of SIX1 in EC cells. Two putative SIX1-binding elements within the CP promoter region were identified based on the JASPAR (http://jaspar.genereg.net/) 33, (**Fig. 6C**). More importantly, we found that SIX1 failed to promote CP transcriptional activity without E1, indicating that E1 is essential for SIX1 to activate CP transcription (**Fig. 6D**). Consistently, ChIP-qPCR assays indicated the enrichment of SIX1 in the promoter of CP (**Fig. 6E**). Overall, those results indicated that SIX1 promotes EC progression via CP-mediated iron homeostasis.

### LINC02936 increases CP expression and suppresses ferroptosis of EC cells interacting with SIX1

**Figure 7:**
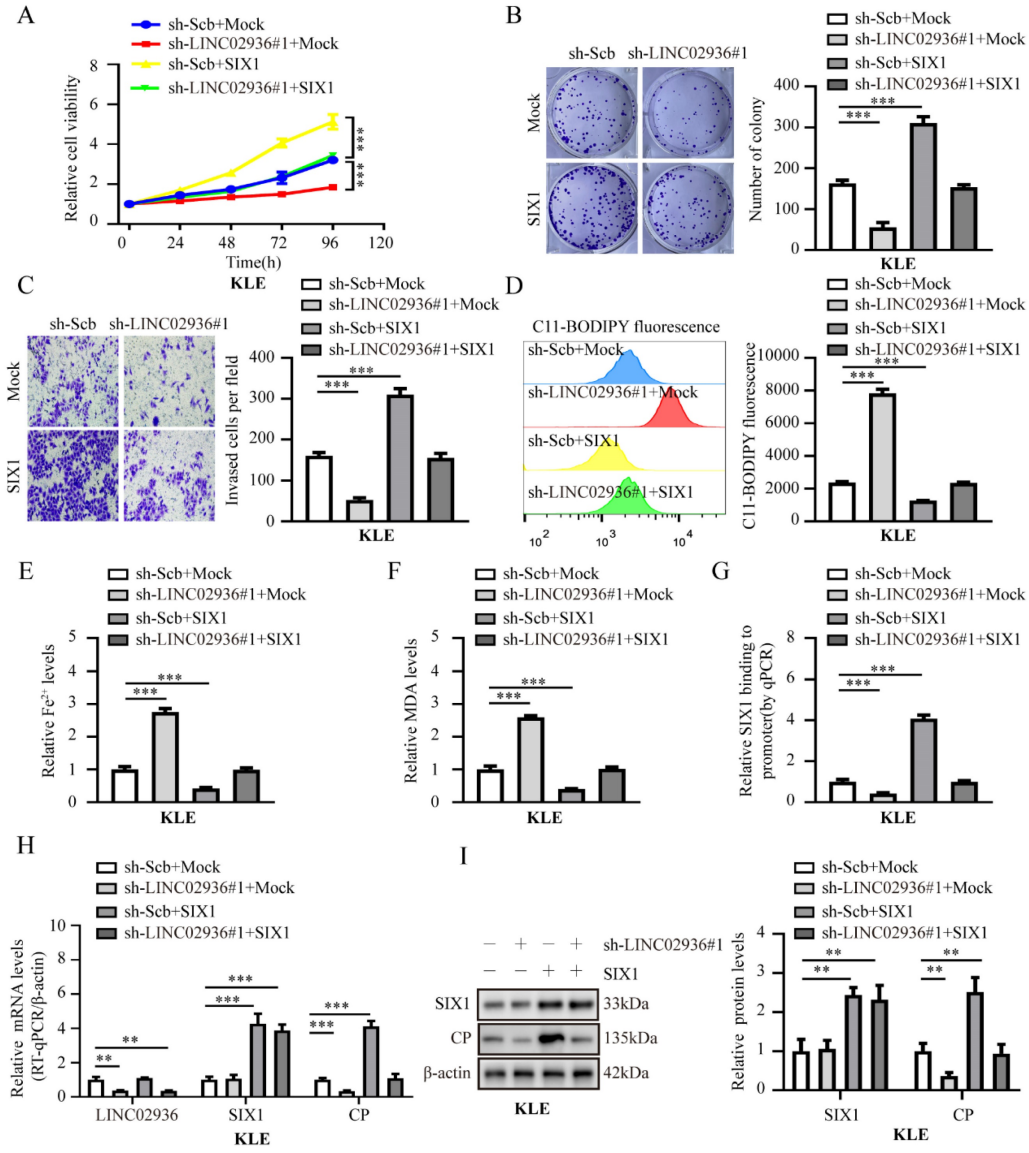
** Silencing of LINC02936 promotes ferroptosis and suppresses tumor progression of EC cells via SIX1‐mediated down-regulation of CP.** (**A-C**) The cell ability (**A**), proliferation (**B**), and invasion (**C**) capacity in KLE cell lines stably transfected with sh‐Scb, sh-LINC02936#1 and those co‐transfected with mock or SIX1, respectively (n = 3). (**D-F**) Relative lipid ROS (**D**), Fe^2+^ (**E**) and MDA (**F**) levels were measured in KLE cells stably transfected with sh‐Scb, sh‐LINC02936#1 and those co‐transfected with mock or CP, respectively (n = 3). (**G**) ChIP assays (normalized to input) suggested the SIX1 enrichment of CP in KLE cells stably transfected with sh-scb, sh-LINC02936#1 and those co‐transfected with mock and SIX1, respectively (n = 3). (**H**) The mRNA levels of LINC02936, SIX1 and CP in KLE cells stably transfected with sh-Scb, sh-LINC02936#1 and those co‐transfected with mock and SIX1 were detected by RT-qPCR, respectively (n = 3). (**I**) The protein levels of SIX1 and CP in KLE cells stably transfected with sh-scb, sh-LINC02936#1 and those co‐transfected with mock and SIX1 by western blot, respectively (n = 3). Post-hoc Bonferroni's test after one-way ANOVA analysis was applied in panel **A-I**. * *P* < 0.05; ** *P* < 0.01; *** *P* < 0.001.

Subsequently, we investigated the regulatory mechanisms of the LINC02936/SIX1/CP axis. Ectopic expression of SIX1 rescued the decreased cell viability, growth, invasion capacity, and decreased the relative lipid ROS, Fe^2+^, and MDA levels induced by stable silencing of LINC02936 in KLE cells (**Fig. 7A-F**). Moreover, knockdown of SIX1 abolished the increased cell viability, growth, invasion capacity, and decreased the relative lipid ROS, Fe^2+^, and MDA levels induced by ectopic LINC02936 in HEC1A cells (**Fig. S7A-F**). In addition, stable enhancement or depletion of LINC02936 elevated and reduced SIX1 enrichment on CP promoters in HEC1A and KLE cells, respectively, which could be rescued by the depletion or enhanced expression of SIX1, respectively (**Fig. 7G**, **Fig. S7G**). Notably, ectopic and knockdown expression of SIX1 abolished CP gene expression induced by the depletion or increased LINC02936 expression in KLE and HEC1A cells, respectively (**Fig. 7H-I**, **Fig. S7H-I**). These findings suggested that LINC02936 increases CP expression and suppresses ferroptosis by interacting with SIX1 in EC cells.

### Therapeutic blocking LINC02936-SIX1 interaction inhibits EC progression by promoting ferroptosis

**Figure 8:**
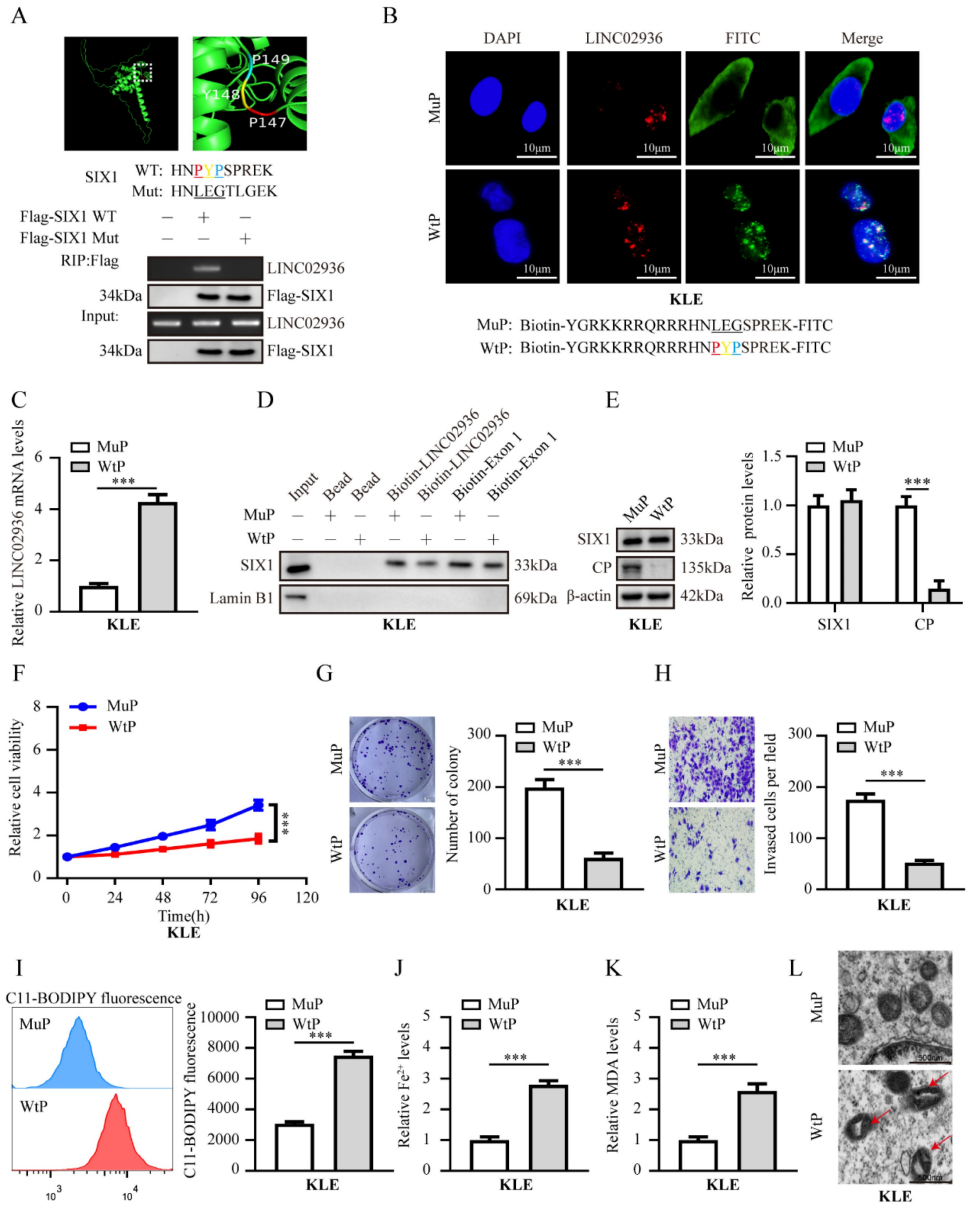
** Therapeutic blocking LINC02936-SIX1 interaction inhibits EC progression by promoting ferroptosis *in vitro*.** (**A**) The potential RNA-binding domain of SIX1 displayed by Pymol software (up panel). The RIP experiment revealed the interaction between Flag‐tagged wild-type (WT) or mutant (Mut) SIX1 and LINC02936 in KLE cells. (**B**) Confocal images revealed the distribution and co‐localization of in KLE cells after treatment 48 h with FITC‐labeled wild type peptide (WtP) or mutant peptide (MuP) (30 µmol L^-1^) and LINC02936 (red). Scale bars: 10 µm. (**C**) The levels of LINC02936 pulled down by biotin‐labeled WtP or MuP (30 µmol L^-1^) from KLE cells for 48 h were tested RT‐qPCR (normalized to input) (n = 3). (**D**) Biotin‐labeled RNA pull‐down assay suggested the interaction of LINC02936 with SIX1 in KLE cells treated with WtP or MuP (30 µmol L^-1^). Bead presented as a negative control. (**E**) The relative protein expression of SIX1 and CP in KLE cells treated with WtP or MuP (30 µmol L^-1^) for 48 h (n = 3) were detected by western blot. (**F-H**) The relative cell ability (**F**), proliferation(**G**), and invasion ability (**H**) treated with WtP or MuP (30 µmol L^-1^) for 48 h in KLE cells, respectively (n = 3). (**I-K**) Relative lipid ROS (**I**), Fe^2+^ (**J**) and MDA (**K**) levels were measured in KLE cells treated with WtP or MuP (30 µmol L^-1^) for 48 h, respectively (n = 3). (**L**) The distinctive changes in mitochondrial morphology in KLE cell lines after treatment 48h with WtP or MuP (30 µmol L^-1^) for 48 h. (The red arrow represents the ferroptosis characteristic mitochondria). Student's t test compared the statistical difference in panel **C**, **E**-**K**. * *P* < 0.05; ** *P* < 0.01; *** *P* < 0.001.

**Figure 9:**
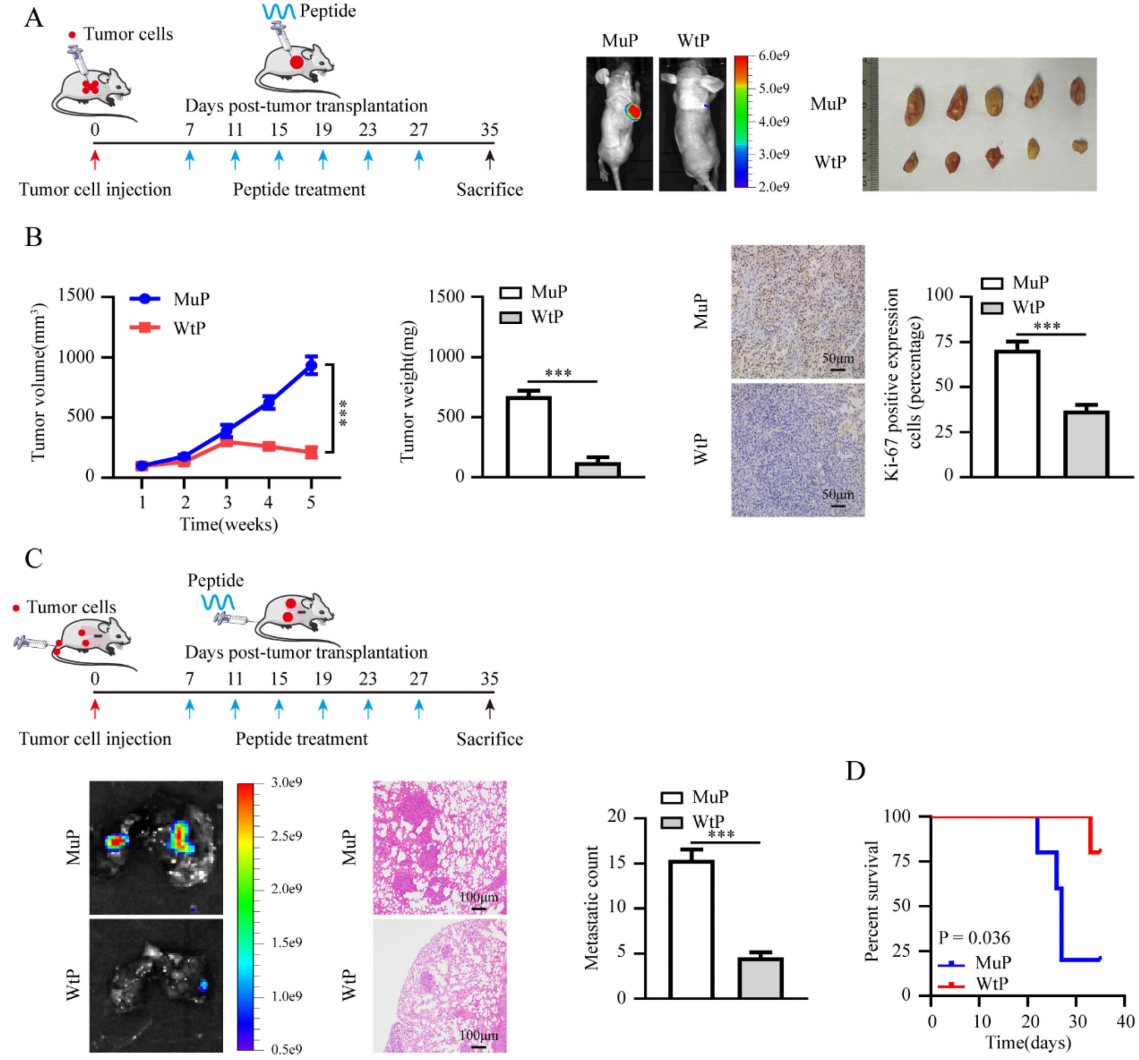
** Therapeutic blocking LINC02936-SIX1 interaction inhibits EC progression *in vivo*.** (**A**) The treatment diagram (left panel) and the representative image of xenograft tumors (right panel) injected KLE cells after treatment with WtP or MuP (3 mg kg^-1^) (n = 5 per group). (**B**) The growth curve (left panel), tumor weight (middle panel) and Ki-67 percentage (right panel, scale bar: 50 μm) of xenograft tumors injected KLE cells after treatment with WtP or MuP (3 mg kg^-1^) (n = 5 per group). (**C**) The treatment diagram (upper panel) and representative lung metastasis images (lower left panel), HE staining images (scale bar: 100 μm) and lung metastatic counts (lower middle panels) and Kaplan-Meier curves (lower right panel) of nude mice injected of KLE cells and treatment with WtP or MuP (3 mg kg^-1^) from tail vein (n = 5 per group). Student's t test compared the statistical difference in panel **B-C**. Survival analysis used Log-rank test in panel **D**. * *P* < 0.05; ** *P* < 0.01; *** *P* < 0.001.

Bioinformatics analysis using using PPRInt 34 and hybridNAP 35 program indicated the potential involvement of 45 residues (124-168 residues of SIX1), particularly three crucial amino acid residues (PYP), within the DNA-binding domain of SIX1 were responsible for binding to LINC02936 (**Fig. 8A**, **Table S5**). Mutations in these three residues eliminated the binding of SIX1 to LINC02936 in KLE cells (**Fig. 8A**). Treatment with cell-penetrating fluorescein isothiocyanate (FITC)-labeled SIX1 inhibiting peptide (WtP) led to nuclear enrichment and co-localization of LINC02936 in KLE cells (**Fig. 8B**). A biotin-labelled peptide pull-down assay revealed the binding of WtP to LINC02936 (**Fig. 8C**). Furthermore, WtP treatment reduced the interaction between SIX1 and LINC02936, resulting in decreased CP expression, cell viability, proliferation, and invasion capacity of KLE cells (**Fig. 8D-H**). Notably, the relative lipid ROS, Fe^2+^, and MDA levels were substantially elevated after WtP treatment (**Fig. 8I-K**). We also confirmed mitochondrial changes in KLE cells after WtP treatment using transmission electron microscopy (**Fig. 8L**). Intratumoral administration of WtP notably reduced tumor growth, weight, and Ki‐67 percentage of subcutaneous xenograft tumors compared to MuP treatment (**Fig. 9A-B**). In addition, treatment with WtP resulted in lower lung metastatic counts and prolonged the survival time of nude mice injected with KLE cells (**Fig. 9C-D**). Together, these findings indicated that blocking the interaction between LINC02936 and SIX1 inhibits EC progression by promoting ferroptosis.

### Elevated expression of LINC02936, SIX1 and CP in human EC tissues

**Figure 10:**
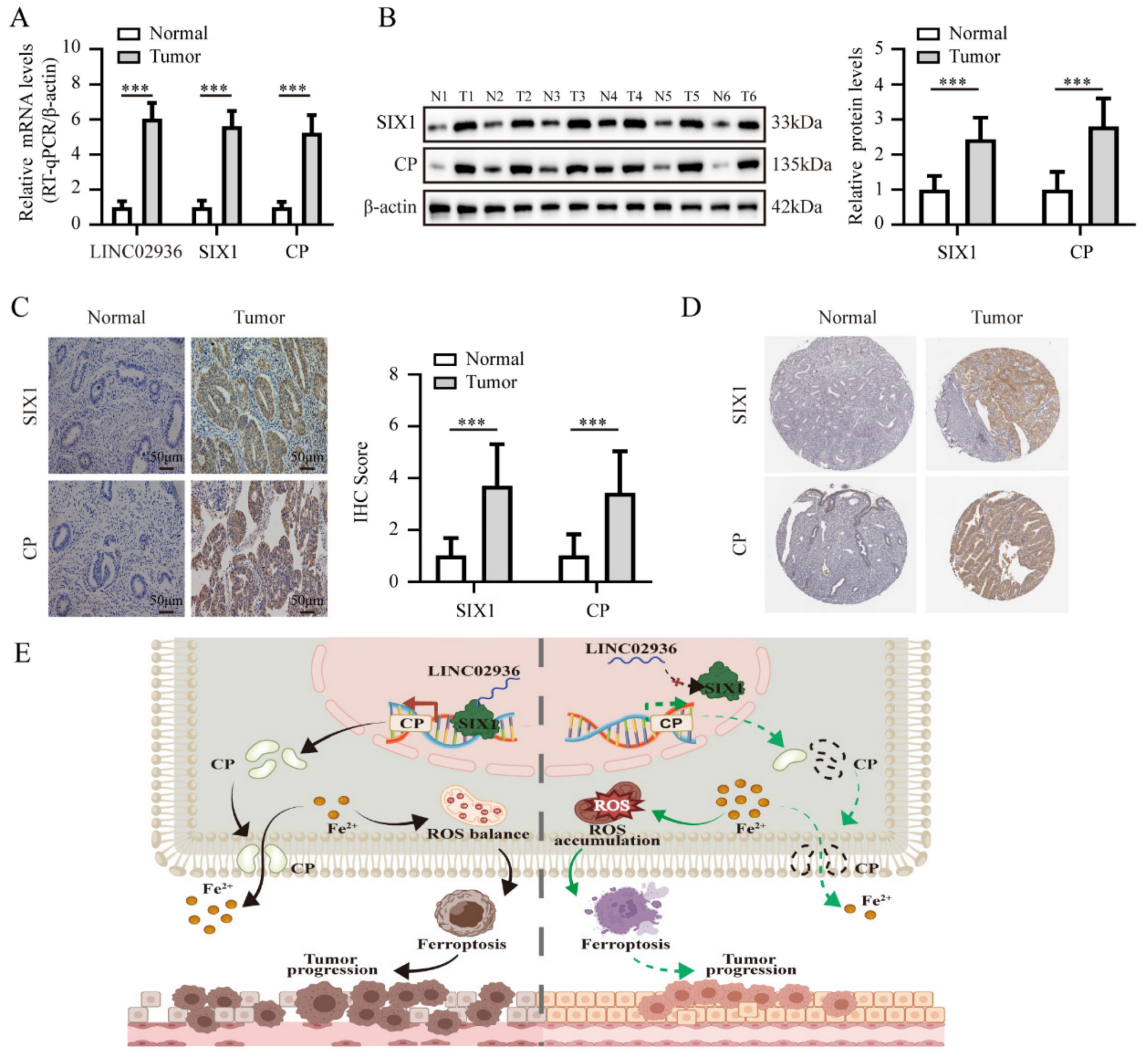
** The expression of LINC02936, SIX1 and CP in human EC tissues.** (**A**) The mRNA levels of LINC02936, SIX1 and CP in EC and matched normal endometrium were tested by RT-qPCR assay (n = 48). (**B**) The protein expression of SIX1 and CP in EC and matched normal endometrium were detected by western blot (n = 48). (**C**) The representative image of IHC indicated the expression of SIX1 and CP in EC and matched normal endometrium (n = 48). (**D**) The representative IHC images of SIX1 and CP in EC and normal endometrial tissues from the Human Protein Atlas (HPA) database (https://www.proteinatlas.org/). (**E**) The mechanisms underlying LINC02936 suppresses ferroptosis and promotes EC progression: SIX1 regulates the expression of CP, while LINC02936 interacts with SIX1 and recruited SIX1 to the promoter regions of CP, resulting in upregulation of CP and suppressing ferroptosis and promotes EC progression. The statistical difference in panel **A-C** was compared using a paired t-test. * *P* < 0.05; ** *P* < 0.01; *** *P* < 0.001.

Subsequently, we conducted further validation to ascertain the significance of LINC02936, SIX1, and CP expression in EC tissues. The RT-qPCR results demonstrated that the mRNA expression of LINC02936, SIX1, and CP exhibited elevated levels in EC compared to normal endometrial tissues (**Fig. 10A**). Subsequent experiments using western blotting and IHC assays also confirmed significantly elevated protein expression levels of SIX1 and CP in EC compared to normal endometrial tissues (**Fig. B-C**). Moreover, the IHC assays indicated a robust positive correlation between the expression of SIX1 and age (χ2=4.057, *P*=0.044) as well as grade (χ2=9.763, *P*=0.002), while no significant associations were observed between SIX1 expression and other clinicopathological features in EC patients, including stage (χ2=0.672, *P*=0.412), histological subtypes (χ2=0.400, *P*=0.527), and lymph node metastasis (χ2=1.769, *P*=0.288) (**Table S6**). Also, the expression level of CP exhibited positive correlations with age (χ2=5.259, *P*=0.022), grade (χ2=6.583, *P*=0.010), and histological subtypes (χ2=6.095, *P*=0.014). However, there was no significant relationship observed between CP expression and other clinicopathological features, including stage (χ2=3.387, *P*=0.066) and lymph node metastasis (χ2=0.676, *P*=0.498) in EC patients (**Table S6**). Our results indicated a consistently and significantly higher expression of SIX1 and CP in the age group of over 60 years and the G3 group (**Table S6**), which were similar to the TCGA-UCEC. Additionally, our findings were consistently with the data in the Human Protein Atlas (HPA) database (**Fig. 10D**). In summary, these data suggested that targeting the LINC02936/SIX1/CP axis may hold promise as an attractive therapeutic approach for treating EC.

## Discussion

Ferroptosis acts as a novel form of programmed cell death mainly characterized by the accumulation of iron and increased lipid peroxide ROS in cells 12. The occurrence of iron death involves several mechanism imbalances, such as glutamate-cysteine antiporter (system XC-), GPX4-GSH and iron transporters 36. The cystine/glutamate antiporter SLC7A11 plays a key effect in the introduction of cystine into the synthesis of glutathione and antioxidant defense and is highly expressed in numerous human cancers 37. GPX4 removes membrane lipid peroxide products and protects the cells from oxidative damage. In addition, GPX4 is considered a potential oncogene that is highly expressed in multiple tumors, including EC 14. In addition, other iron metabolism genes, such as TFRC, HMOX1, HSPB1, and IREB2 are associated with ferroptosis 38, 39. CP is a glycoprotein known for its ferroxidase and amine chlorinase activities, which contribute to its role as an antioxidant by impeding the formation of free radicals 40. Research has indicated that CP acts as a copper transporter in the blood 41. Furthermore, it also plays a crucial role in maintaining iron homeostasis 15, 40. Our study revealed that the iron metabolism gene CP exhibits abnormal expression in EC and is closely linked to a poor prognosis, potentially linked to the suppression of ferroptosis. Other studies reported similar results 15, 16, 19. These findings suggested that CP may represent a potential target for ferroptosis in EC.

CP is a multicopper oxidase and ferroxidase that crucially maintains the metabolic equilibrium between copper and iron 42. Numerous literatures have proposed that anomalous expression of CP is associated with various diseases. For instance, CP-knockout mice have impaired behavioral abilities through iron accumulation, and CP replacement therapy can improve these symptoms 43. Serum CP is significantly increased in preeclampsia and may be a potential indicator for the evaluation of pregnant women with a history of preeclampsia 44. Anomalous CP expression is linked with tumor development and progression. Increased expression of ceruloplasmin (CP) is associated with an unfavorable prognosis and immune infiltration in breast cancer 45. Another study indicated a positive association between CP and the progression of bladder cancer 17. Additionally, the expression of ceruloplasmin (CP) was significantly linked to high-grade and a worse prognosis in clear cell renal cell carcinoma 46, which is similar to other EC study 19 and our results. However, CP was lowly expressed in other cancers, such as nasopharyngeal carcinoma 47. More importantly, multiple mechanisms have been shown to promote or inhibit disease progression by regulating CP expression. LINC00176 promotes ovarian cancer progression through the BCl3-mediated upregulation of CP 28. SARI promotes colon cancer progression and angiogenesis by upregulating CP and HIF-1α/VEGF axis 48. miR-543 suppresses tumor progression by negatively regulating CP 47. However, although CP is abnormally expressed and related to poor prognosis in EC, the mechanism of its regulation is not understood. Our results Our findings indicated that overexpression of CP decreases Fe^2+^ and lipid ROS levels, suppressing ferroptosis and promoting malignant progression of EC, consistent with previous studies 15, 16.

Long non-coding RNAs (lncRNAs) are a subclass of non-coding RNAs (ncRNAs) that exceed 200 nucleotides in length. Previous literature have revealed that lncRNAs can either inhibit or promote tumor progression by regulating the ferroptosis genes expression. For example, the lncRNA OIP5-AS1 suppresses ferroptosis and promotes tumor malignant progression in prostate cancer by activating the miR-128-3p/SLC7A11 pathway 49. Moreover, lncRNA HEPFAL contributes ferroptosis and suppresses tumor progression by affecting SLC7A11 ubiquitination in hepatocellular carcinoma 50. LINC00239 suppresses ferroptosis and promotes cancer progression in colorectal cancer by interacting with Keap1 to stabilize Nrf2 51. Although lncRNA are abnormally expressed in EC and are closely correlated to tumor development and chemotherapy resistance 52, no studies have reported on lncRNAs promoting EC progression by regulating ferroptosis. To the best of our understanding, this research is the first literature to explore the precise molecular mechanism by which lncRNAs regulate ferroptosis and advance the malignant progression of EC. Our study revealed that LINC02936 promotes tumor progression and suppresses ferroptosis in EC by binding to SIX1 and upregulating CP expression. The transcription factor SIX1, a member of the SIX family, is closely associated with the progression of cancer and portends a poor prognosis in various malignancies 53. SIX1 is also widely involved in tumor metabolism, chemotherapy resistance, and immune escape processes 54, 55. In addition, SIX1 is overexpressed in EC and linked to an unfavorable clinicopathological outcome 56, which is consistent with our findings. However, the precise molecular mechanism through which SIX1 promotes EC progression by modulating iron homeostasis remains unclear. Our research filled this gap in the literature by introducing SIX1, which increases CP expression and suppresses ferroptosis.

However, our study has several limitations. The effects of ferritin inhibitors and activators on LINC02936 and SIX1 expression have not been extensively explored. Given the critical role of CP in copper transport, it is unclear whether abnormal CP expression promotes tumor progression by reprogramming copper metabolism. Despite previous research suggesting that copper supplementation cannot reverse CP-mediated ferroptosis in hepatocellular carcinoma cells 15, whether CP is involved in cuproptosis needs to be further explored.

In summary, we found that LINC02936, the TF SIX1, and its target gene CP are highly expressed and related to poorer outcomes in patients with EC, and act as an oncogenic role in regulating ferroptosis and tumor progression. Mechanistically, SIX1 modulates the expression of CP, whereas LINC02936 interacts with SIX1 and recruits SIX1 to the promoter of CP, leading to the upregulation of CP and inhibition of ferroptosis, which promotes EC progression. The research broadens the comprehension of lncRNA-mediated regulation of ferroptosis in EC progression, suggesting the potential therapeutic significance of the LINC02936/SIX1/CP axis in treating EC.

## Supplementary Material

Supplementary figures and tables.

## References

[1] Crosbie EJ, Kitson SJ, McAlpine JN, Mukhopadhyay A, Powell ME, Singh N (2022). Endometrial cancer. Lancet.

[2] Sobel M, Simpson AN, Ferguson SE (2021). Endometrial cancer. CMAJ.

[3] Reeves GK, Pirie K, Beral V, Green J, Spencer E, Bull D (2007). Cancer incidence and mortality in relation to body mass index in the Million Women Study: cohort study. BMJ.

[4] Tokmak A, Kokanali MK, Guzel AI, Kara A, Topcu HO, Cavkaytar S (2014). Polycystic ovary syndrome and risk of endometrial cancer: a mini-review. Asian Pac J Cancer Prev.

[5] Chifman J, Laubenbacher R, Torti SV (2014). A systems biology approach to iron metabolism. Adv Exp Med Biol.

[6] Hsu MY, Mina E, Roetto A, Porporato PE (2020). Iron: An Essential Element of Cancer Metabolism. Cells.

[7] Daniels TR, Delgado T, Rodriguez JA, Helguera G, Penichet ML (2006). The transferrin receptor part I: Biology and targeting with cytotoxic antibodies for the treatment of cancer. Clin Immunol.

[8] Tortorella S, Karagiannis TC (2014). Transferrin receptor-mediated endocytosis: a useful target for cancer therapy. J Membr Biol.

[9] Walker RA, Day SJ (1986). Transferrin receptor expression in non-malignant and malignant human breast tissue. J Pathol.

[10] Prior R, Reifenberger G, Wechsler W (1990). Transferrin receptor expression in tumours of the human nervous system: relation to tumour type, grading and tumour growth fraction. Virchows Arch A Pathol Anat Histopathol.

[11] Kondo K, Noguchi M, Mukai K, Matsuno Y, Sato Y, Shimosato Y (1990). Transferrin Receptor Expression in Adenocarcinoma of the Lung as a Histopathologic Indicator of Prognosis. Chest.

[12] Xu T, Ding W, Ji X, Ao X, Liu Y, Yu W (2019). Molecular mechanisms of ferroptosis and its role in cancer therapy. J Cell Mol Med.

[13] Yan J, Ye G, Shao Y (2022). High expression of the ferroptosis-associated MGST1 gene in relation to poor outcome and maladjusted immune cell infiltration in uterine corpus endometrial carcinoma. J Clin Lab Anal.

[14] Wei S, Yu Z, Shi R, An L, Zhang Q, Zhang Q (2022). GPX4 suppresses ferroptosis to promote malignant progression of endometrial carcinoma via transcriptional activation by ELK1. BMC Cancer.

[15] Shang Y, Luo M, Yao F, Wang S, Yuan Z, Yang Y (2020). Ceruloplasmin suppresses ferroptosis by regulating iron homeostasis in hepatocellular carcinoma cells. Cell Signal.

[16] Yang M, Wu X, Hu J, Wang Y, Wang Y, Zhang L (2022). COMMD10 inhibits HIF1alpha/CP loop to enhance ferroptosis and radiosensitivity by disrupting Cu-Fe balance in hepatocellular carcinoma. J Hepatol.

[17] Mukae Y, Ito H, Miyata Y, Araki K, Matsuda T, Aibara N (2021). Ceruloplasmin Levels in Cancer Tissues and Urine Are Significant Biomarkers of Pathological Features and Outcome in Bladder Cancer. Anticancer Res.

[18] Han IW, Jang JY, Kwon W, Park T, Kim Y, Lee KB (2017). Ceruloplasmin as a prognostic marker in patients with bile duct cancer. Oncotarget.

[19] Gusarova Iu N, Stepanova EV, Landesman EO, Koroleva OV, Vavilova TP, Makarov OV (2002). [Regulation of ceruloplasmin activity in oncogynecological diseases]. Vopr Med Khim.

[20] Zhou N, Bao J (2020). FerrDb: a manually curated resource for regulators and markers of ferroptosis and ferroptosis-disease associations. Database (Oxford). 2020.

[21] Zhang Z, Chen F, Li S, Guo H, Xi H, Deng J (2020). ERG the modulates Warburg effect and tumor progression in cervical cancer. Biochem Biophys Res Commun.

[22] Zhang Z, Li S, Deng J, Yang S, Xiang Z, Guo H (2020). Aspirin inhibits endometrial fibrosis by suppressing the TGF-beta1-Smad2/Smad3 pathway in intrauterine adhesions. Int J Mol Med.

[23] Passarello K, Kurian S, Villanueva V (2019). Endometrial Cancer: An Overview of Pathophysiology, Management, and Care. Semin Oncol Nurs.

[24] Weijiao Y, Fuchun L, Mengjie C, Xiaoqing Q, Hao L, Yuan L (2021). Immune infiltration and a ferroptosis-associated gene signature for predicting the prognosis of patients with endometrial cancer. Aging (Albany NY).

[25] Park SJ, Yoon BH, Kim SK, Kim SY (2019). GENT2: an updated gene expression database for normal and tumor tissues. BMC Med Genomics.

[26] Valanezhad A, Odatsu T, Abe S, Watanabe I (2021). Bone Formation Ability and Cell Viability Enhancement of MC3T3-E1 Cells by Ferrostatin-1 a Ferroptosis Inhibitor of Cancer Cells. Int J Mol Sci.

[27] Qu L, He X, Tang Q, Fan X, Liu J, Lin A (2022). Iron metabolism, ferroptosis, and lncRNA in cancer: knowns and unknowns. J Zhejiang Univ Sci B.

[28] Dai L, Niu J, Feng Y (2020). Knockdown of long non-coding RNA LINC00176 suppresses ovarian cancer progression by BCL3-mediated down-regulation of ceruloplasmin. J Cell Mol Med.

[29] Kang YJ, Yang DC, Kong L, Hou M, Meng YQ, Wei L (2017). CPC2: a fast and accurate coding potential calculator based on sequence intrinsic features. Nucleic Acids Res.

[30] Cui T, Dou Y, Tan P, Ni Z, Liu T, Wang D (2022). RNALocate v2.0: an updated resource for RNA subcellular localization with increased coverage and annotation. Nucleic Acids Res.

[31] Dangelmaier E, Lal A (2020). Adaptor proteins in long noncoding RNA biology. Biochim Biophys Acta Gene Regul Mech.

[32] Muppirala UK, Honavar VG, Dobbs D (2011). Predicting RNA-protein interactions using only sequence information. BMC Bioinformatics.

[33] Castro-Mondragon JA, Riudavets-Puig R, Rauluseviciute I, Lemma RB, Turchi L, Blanc-Mathieu R (2022). JASPAR 2022: the 9th release of the open-access database of transcription factor binding profiles. Nucleic Acids Res.

[34] Kumar M, Gromiha MM, Raghava GP (2008). Prediction of RNA binding sites in a protein using SVM and PSSM profile. Proteins.

[35] Zhang J, Ma Z, Kurgan L (2019). Comprehensive review and empirical analysis of hallmarks of DNA-, RNA- and protein-binding residues in protein chains. Brief Bioinform.

[36] Lei G, Zhuang L, Gan B (2022). Targeting ferroptosis as a vulnerability in cancer. Nat Rev Cancer.

[37] Koppula P, Zhuang L, Gan B (2021). Cystine transporter SLC7A11/xCT in cancer: ferroptosis, nutrient dependency, and cancer therapy. Protein Cell.

[38] Bogdan AR, Miyazawa M, Hashimoto K, Tsuji Y (2016). Regulators of Iron Homeostasis: New Players in Metabolism, Cell Death, and Disease. Trends Biochem Sci.

[39] Hassannia B, Vandenabeele P, Vanden Berghe T (2019). Targeting Ferroptosis to Iron Out Cancer. Cancer Cell.

[40] Musci G, Polticelli F, Bonaccorsi di Patti MC (2014). Ceruloplasmin-ferroportin system of iron traffic in vertebrates. World J Biol Chem.

[41] Marques L, Auriac A, Willemetz A, Banha J, Silva B, Canonne-Hergaux F (2012). Immune cells and hepatocytes express glycosylphosphatidylinositol-anchored ceruloplasmin at their cell surface. Blood Cells Mol Dis.

[42] Liu Z, Wang M, Zhang C, Zhou S, Ji G (2022). Molecular Functions of Ceruloplasmin in Metabolic Disease Pathology. Diabetes Metab Syndr Obes.

[43] Niu L, Zhou Y, Lu L, Su A, Guo X (2022). Ceruloplasmin Deficiency Impaired Brain Iron Metabolism and Behavior in Mice. Cell Biochem Biophys.

[44] Bellos I, Papantoniou N, Pergialiotis V (2018). Serum ceruloplasmin levels in preeclampsia: a meta-analysis. J Matern Fetal Neonatal Med.

[45] Chen F, Han B, Meng Y, Han Y, Liu B, Zhang B (2021). Ceruloplasmin correlates with immune infiltration and serves as a prognostic biomarker in breast cancer. Aging (Albany NY).

[46] Zimpfer A, Glass A, Bastian M, Schuff-Werner P, Hakenberg OW, Maruschke M (2021). Ceruloplasmin expression in renal cell carcinoma correlates with higher-grade and shortened survival. Biomark Med.

[47] Yang H, Bao Y, Jin F, Jiang C, Wei Z, Liu Z (2022). Ceruloplasmin inhibits the proliferation, migration and invasion of nasopharyngeal carcinoma cells and is negatively regulated by miR-543. Nucleosides Nucleotides Nucleic Acids.

[48] Dai L, Cui X, Zhang X, Cheng L, Liu Y, Yang Y (2016). SARI inhibits angiogenesis and tumour growth of human colon cancer through directly targeting ceruloplasmin. Nat Commun.

[49] Zhang Y, Guo S, Wang S, Li X, Hou D, Li H (2021). LncRNA OIP5-AS1 inhibits ferroptosis in prostate cancer with long-term cadmium exposure through miR-128-3p/SLC7A11 signaling. Ecotoxicol Environ Saf.

[50] Zhang B, Bao W, Zhang S, Chen B, Zhou X, Zhao J (2022). LncRNA HEPFAL accelerates ferroptosis in hepatocellular carcinoma by regulating SLC7A11 ubiquitination. Cell Death Dis.

[51] Hu J, Lai C, Shen Z, Yu H, Lin J, Xie W (2022). A Prognostic Model of Bladder Cancer Based on Metabolism-Related Long Non-Coding RNAs. Front Oncol.

[52] Liu H, Wan J, Chu J (2019). Long non-coding RNAs and endometrial cancer. Biomed Pharmacother.

[53] Zhu G, Liu Y, Zhao L, Lin ZH, Piao YS (2021). The Significance of SIX1 as a Prognostic Biomarker for Survival Outcome in Various Cancer Patients: A Systematic Review and Meta-Analysis. Front Oncol.

[54] Yang C, Xu W, Gong J, Chai F, Cui D, Liu Z (2020). Six1 Overexpression Promotes Glucose Metabolism and Invasion Through Regulation of GLUT3, MMP2 and Snail in Thyroid Cancer Cells. Onco Targets Ther.

[55] Liu W, Gao M, Li L, Chen Y, Fan H, Cai Q (2021). Homeoprotein SIX1 compromises antitumor immunity through TGF-beta-mediated regulation of collagens. Cell Mol Immunol.

[56] Li W, Qin Y, Zhou R, Liu Y, Zhang G (2021). High expression of SIX1 is an independent predictor of poor prognosis in endometrial cancer. Am J Transl Res.

