# Supplementary material for: Novel LncRNA LINC02936 Suppresses Ferroptosis and Promotes Tumor Progression by Interacting with SIX1/CP Axis in Endometrial Cancer
Source: Int J Biol Sci. 2024 Jan 27;20(4):1356–74. doi: 10.7150/ijbs.86256 (PMC10878161; doi:10.7150/ijbs.86256)
Supplement: Supplementary file 1 — Supplementary figures and tables. [file ijbsv20p1356s1.pdf]

Supplementary Figures and Tables

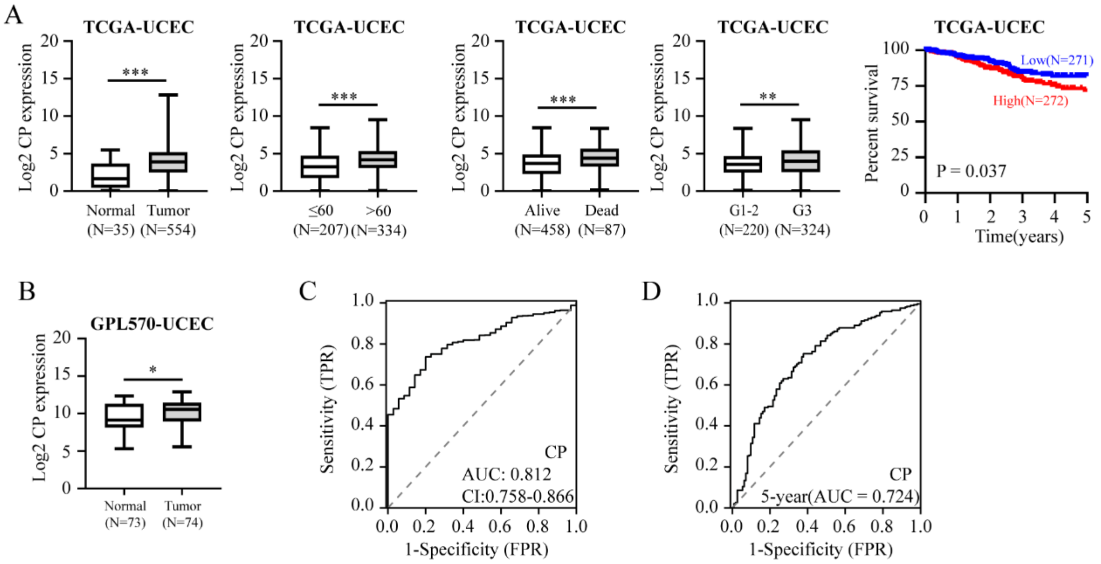

Figure S1 CP exhibits high expression in EC tissures and is associated with an unfavorable prognosis. (**A**) CP was highly expressed in EC tissues, >60 years, G3, dead group and with poor survival in TCGA-UCEC database. (**B**) CP was extremely expressed in EC compare with normal endometrium tissue in GENT2 database. (C-D) ROC curves for the diagnostic (**C**) and prognostic (**D**) capability of CP based on the TCGA-UCEC, respectively. The difference in panel A-B was compared using a Student's t-test. Log-rank test was applied for survival analysis. * $P < 0.05$; ** $P < 0.01$; *** $P < 0.001$.

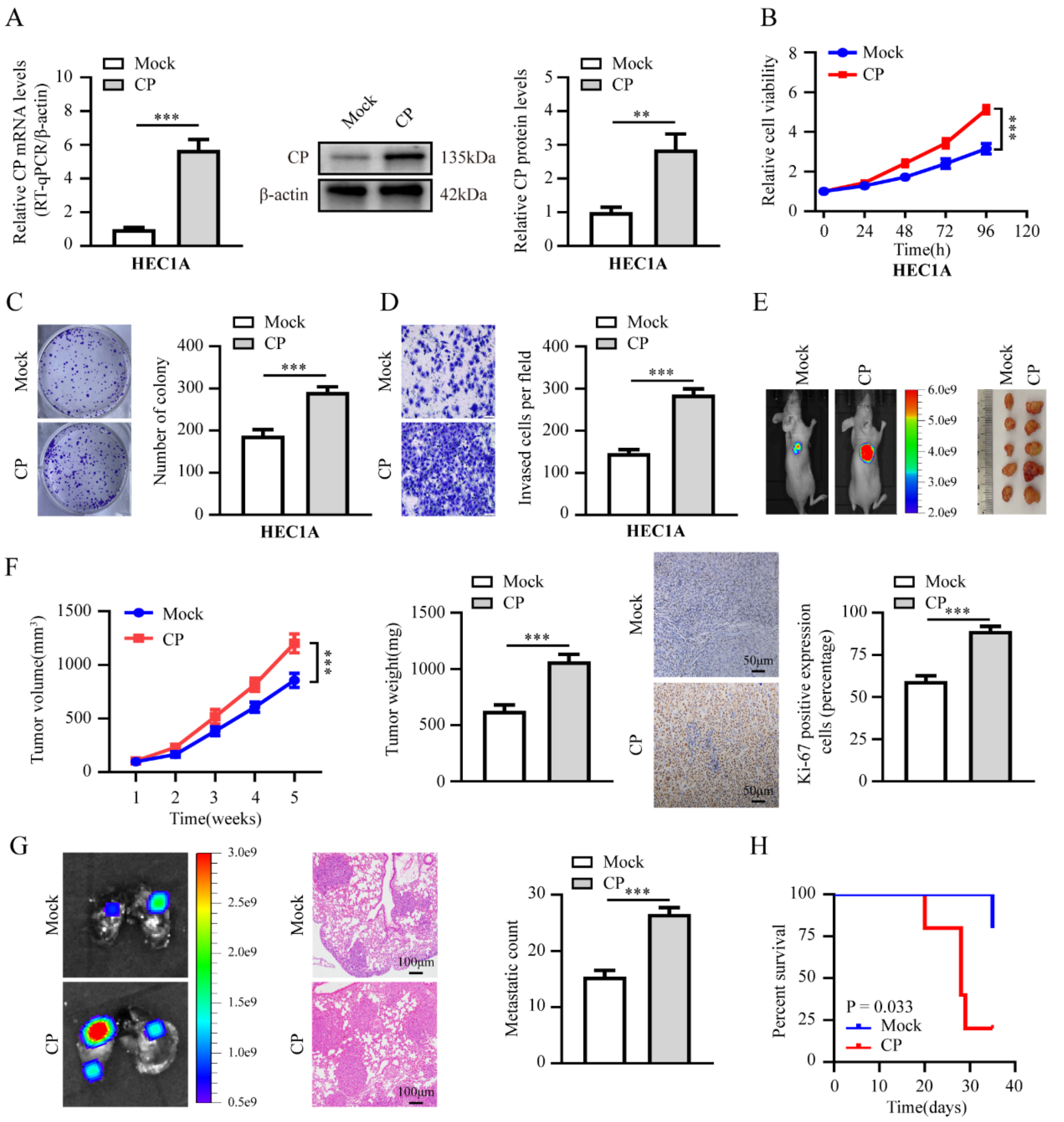

Figure S2 CP promotes tumor progression in HEC1A cells. (**A**) RT-qPCR (left panel) and western blot (right panel) revealed the mRNA and protein expression levels of CP in HEC1A cells stably transfected with mock and CP (n = 3). (**B-D**) The cell ability (**B**), proliferation (**C**), and invasion ability (**D**) in HEC1A cell lines stably transfected with mock and CP, respectively (n = 3). (**E-F**) Representative images of xenograft tumors (**E**), tumor growth (left panel, **F**), weight (middle panel, **F**), Ki-67 percentage (right panel, scale bar: 50 μm, **F**) in nude mice injected HEC1A cells with stably transfected with mock and CP (n = 5 per group), respectively. (**G**) Representative images of lungs

(left panel, scale bar: 100 μm), metastatic counts (right panel) in nude mice injected HEC1A cells into the caudal vein with stably transfected with mock and CP (n = 5 per group), respectively. (**H**) Kaplan-Meier curves suggested the survival time of nude mice in experimental lung metastasis assay (n = 5 per group). The difference in panel **A-G** was compared using a Student's t-test. Log-rank test was applied for survival analysis. * $P < 0.05$; ** $P < 0.01$; *** $P < 0.001$.

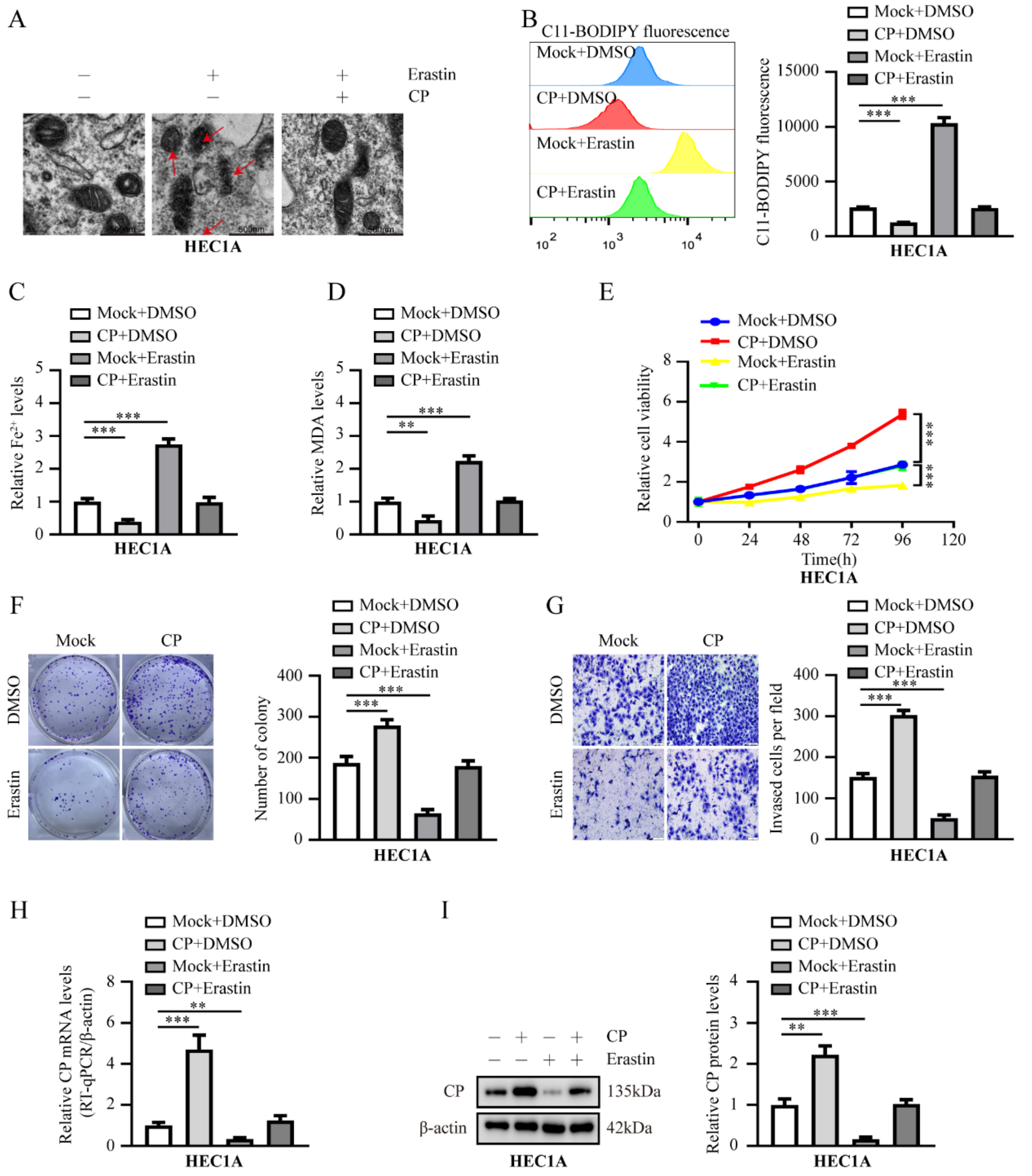

Figure S3 Overexpression of CP promotes EC tumor progression by suppressing

ferroptosis in HEC1A cells. (**A**) The distinctive changes in mitochondrial morphology in HEC1A cell lines after treatment with mock combine with DMSO, mock combine with Erastin (20 μM) or CP combine with Erastin (20 μM), respectively. Scale bar, 500 nm. (The red arrow represents the ferroptosis characteristic mitochondria). (**B-D**) Relative lipid ROS (**B**), Fe^{2+} (**C**) and MDA (**D**) levels were measured in HEC1A cells stably transfected with mock, CP and those co-treatment with DMSO, Erastin (20 μM), respectively (n = 3). (**E-G**) The cell ability (**E**), proliferation (**F**), and invasion capacity (**G**) in HEC1A cell stably transfected with mock, CP and those co-treatment with DMSO, Erastin (20 μM), respectively (n = 3). (**H-I**) The mRNA and protein levels of CP in HEC1A cells stably transfected with mock, CP and those co-treatment with DMSO, Erastin (20 μM) were detected by RT-qPCR (**H**, left panel) and western blot, respectively (n = 3). Post-hoc Bonferroni's test after one-way ANOVA analysis was applied in panel **B-I**. * $P < 0.05$; ** $P < 0.01$; *** $P < 0.001$.

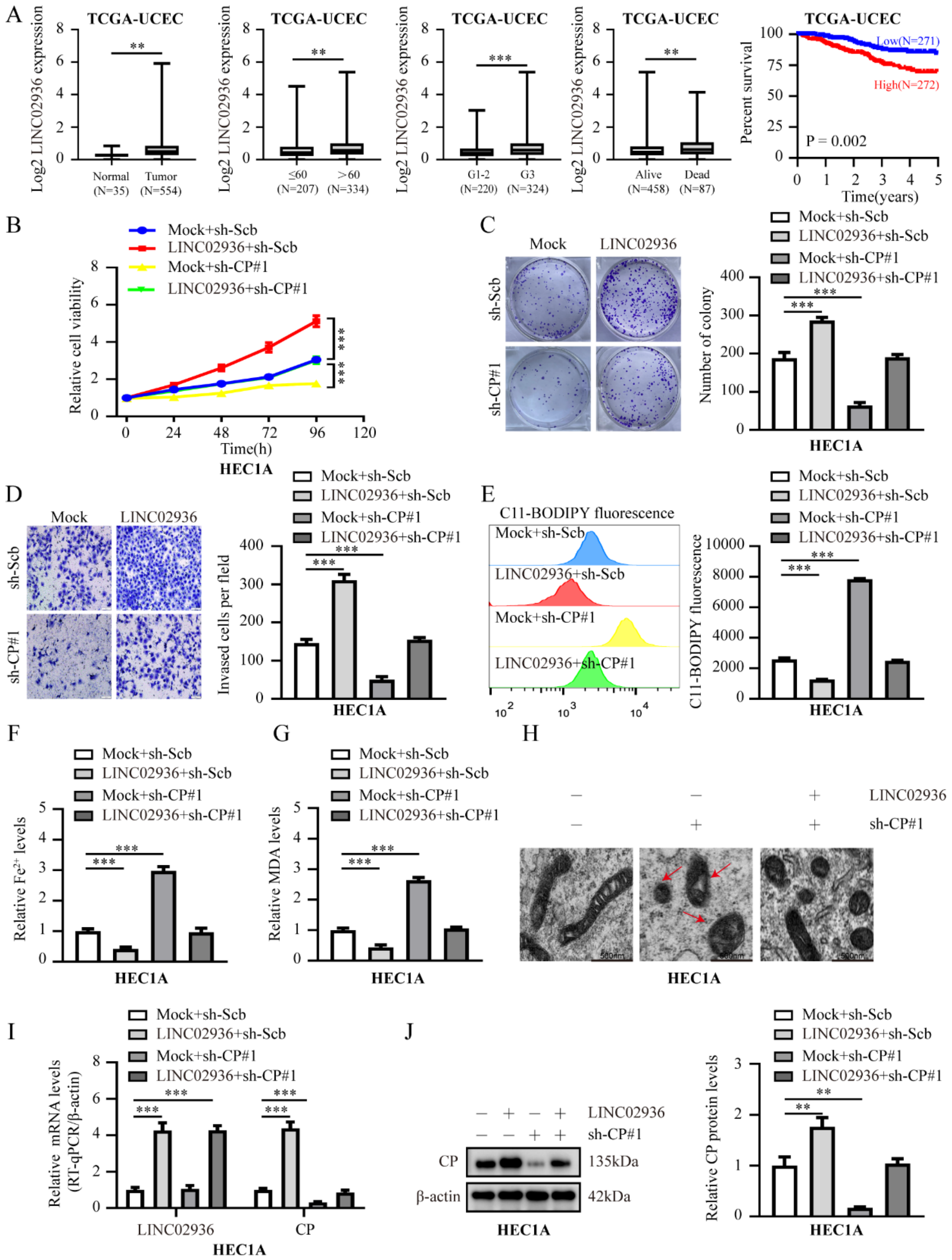

Figure S4 Overexpression of LINC02936 promotes tumor progression by CP-mediated ferroptosis in HEC1A cells. (**A**) LINC02936 was highly expressed in EC tissues, >60 years, G3, dead group and with poor survival in TCGA-UCEC database. (**B-D**) The cell ability (**B**), proliferation(**C**), and invasion ability (**D**) in HEC1A cells

stably transfected with mock, LINC02936 and those co-transfected with sh-Scb or sh-CP#1, respectively (n = 3). (**E-G**) Relative lipid ROS (**E**), Fe^{2+} (**F**) and MDA (**G**) levels were measured in HEC1A cells stably transfected mock, LINC02936 and those co-transfected with sh-Scb or sh-CP#1, respectively (n = 3). (**H**) The distinctive changes in mitochondrial morphology in HEC1A cell lines after treatment with mock sh-Scb combine with mock, sh-CP#1 combine with mcok, sh-CP#1 combine with LINC02936, respectively. Scale bar, 500 nm. (The red arrow represents the ferroptosis characteristic mitochondria). (**I**) Relative mRNA expression of LINC02936 and CP in HEC1A cells stably transfected with mock and LINC02936 by RT-qPCR (n = 3). (**J**) Relative protein expression of CP in HEC1A cell stably transfected with mock, LINC02936 and those co-transfected with sh-Scb or sh-CP#1 by western blot (n = 3). Student's t-test was used to compare the statistical difference in panel **A**. Log-rank test was employed to compare survival rates. Post-hoc Bonferroni's test after one-way ANOVA analysis was applied in panel **B-G and I-J**. * $P < 0.05$; ** $P < 0.01$; *** $P < 0.001$.

A

TFs	lncRNA	RF value	SVM value
CEBPG	LINC02936	0.70	0.81
GLIS2	LINC02936	0.75	0.95
IKZF3	LINC02936	0.70	0.96
MEOX1	LINC02936	0.90	0.95
MESP2	LINC02936	0.75	0.92
SIX1	LINC02936	0.75	0.94
SIX3	LINC02936	0.60	0.91
ZNF516	LINC02936	0.70	0.97

B

TFs	Gene	Cor	p value
CEBPG	CP	0.028	0.518
GLIS2	CP	0.032	0.463
IKZF3	CP	-0.010	0.816
MESP2	CP	0.008	0.845
MEOX1	CP	-0.060	0.159
SIX1	CP	0.154	<0.001
SIX3	CP	-0.011	0.793
ZNF516	CP	-0.084	0.049

C

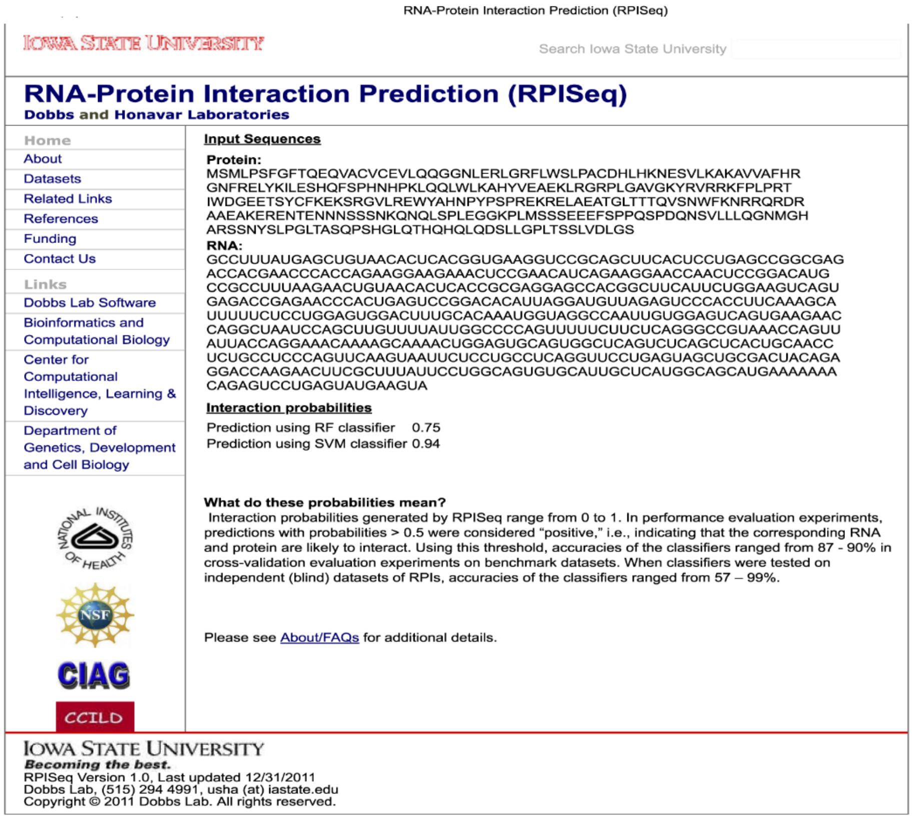

D

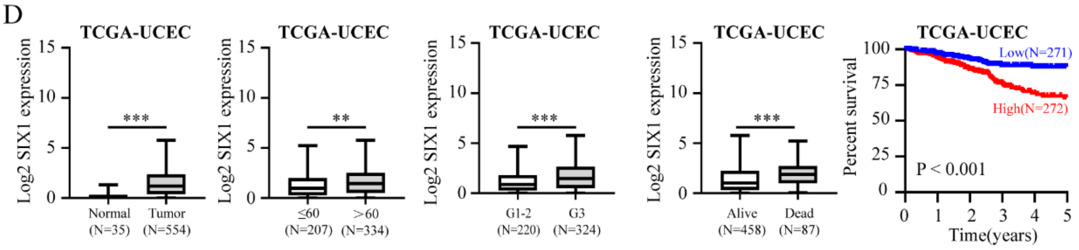

Figure S5 The interaction probability between LINC02936 and eight TFs. (**A**) The RF value and SVM value between LINC02936 and eight TFs were predicted from RPISeq (http://pridb.gdcb.iastate.edu/RPISeq/). (**B**) The correlation analysis between eight TFs and CP based on TCGA-UCEC database. (**C**) The potential interaction

between LINC02936 and SIX1 based on RPISeq program (http://pridb.gdcb.iastate.edu/RPISeq/). (**D**) SIX1 was highly expressed in EC tissues, >60 years, G3, dead group and with poor survival in TCGA-UCEC database. Pearson correlation analysis was used in panel **B**. Student's t-test was used to compare the statistical difference in panel **D**. Log-rank test was employed to compare survival rates. * $P < 0.05$; ** $P < 0.01$; *** $P < 0.001$.

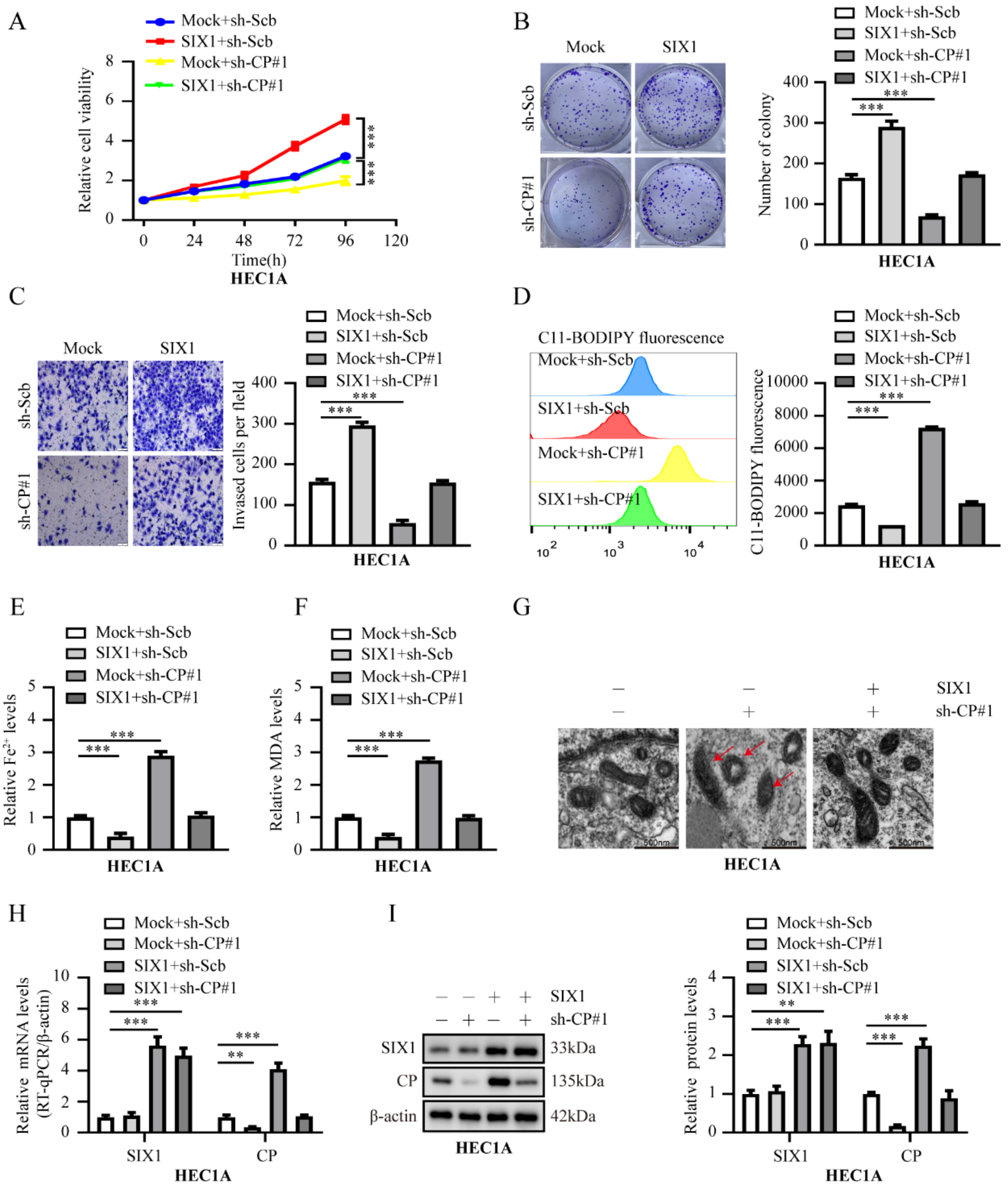

Figure S6 Overexpression of SIX1 promotes EC tumor progression by CP-mediated ferroptosis in HEC1A cells. (**A-C**) The cell ability (**A**), proliferation(**B**), and invasion ability (**C**) in HEC1A cell lines stably transfected with mock, SIX1 and those co-transfected with sh-Scb or sh-CP#1, respectively (n = 3). (**D-F**) Relative lipid ROS (**D**), Fe^{2+} (**E**) and MDA (**F**) levels were measured in HEC1A cells stably transfected with mock, SIX1 and those co-transfected with sh-Scb or sh-CP#1, respectively (n = 3). (**G**) The distinctive changes in mitochondrial morphology in HEC1A cell lines after treatment with mock combine with sh-Scb, mock combine with sh-CP#1, SIX1 combine with sh-CP#1, respectively. Scale bar, 500 nm. (The red arrow represents the ferroptosis characteristic mitochondria). (**H-I**) RT-qPCR (**H**, left panel) and western blot (**I**, right panel) showing the levels of SIX1 and CP in HEC1A cells stably transfected with mock, SIX1 and those co-transfected with sh-Scb and sh-CP#1(n = 3). Post-hoc Bonferroni's test after one-way ANOVA analysis was applied in panel **A-F** and **H-I**. * $P < 0.05$; ** $P < 0.01$; *** $P < 0.001$.

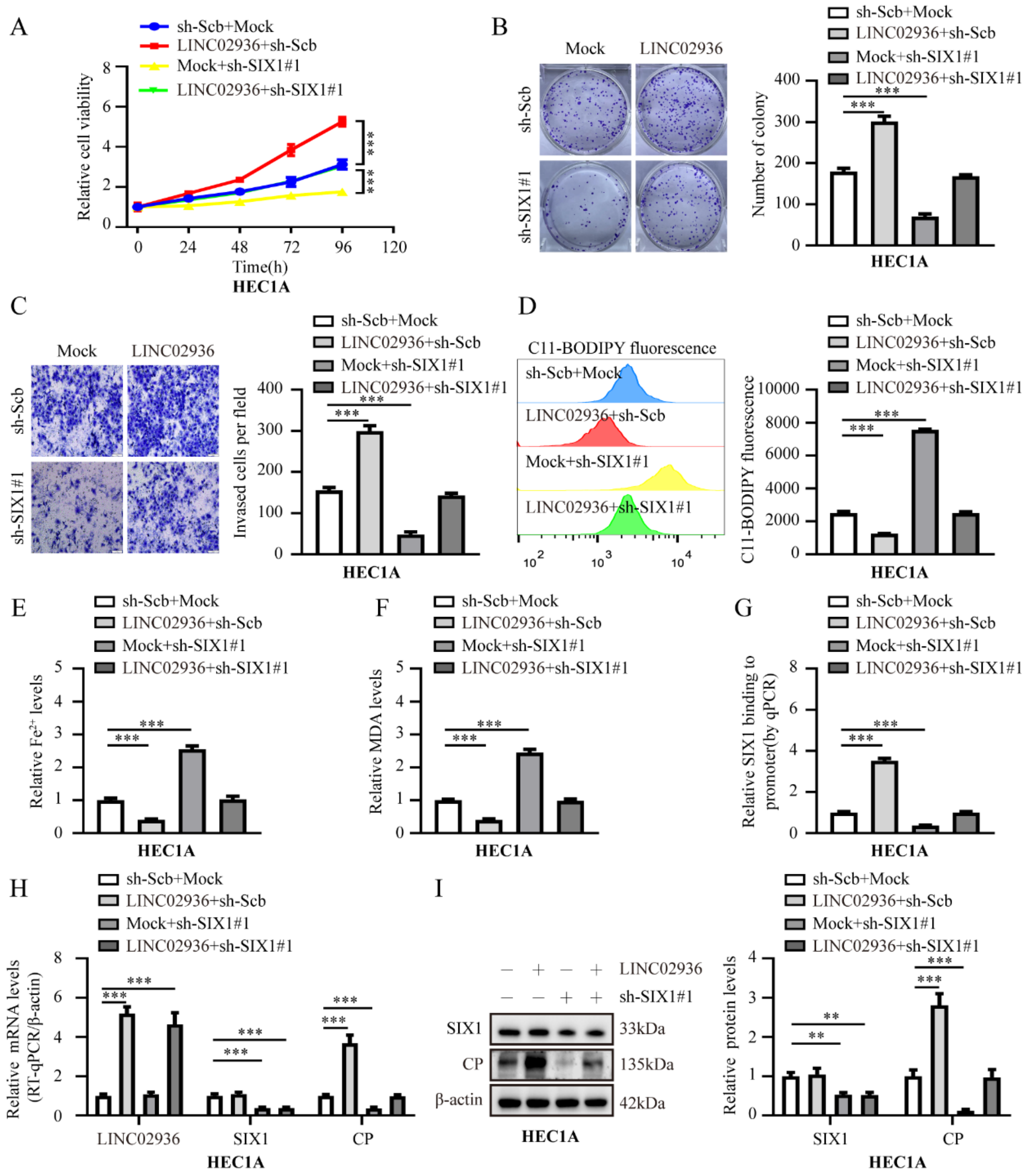

Figure S7 Overexpression of LINC02936 promotes tumor progression and suppresses ferroptosis of EC cells via SIX1-mediated up-regulation of CP in HEC1A cells. (**A-C**) The cell ability (**A**), proliferation (**B**), and invasion ability (**C**) in HEC1A cell lines stably transfected with mock, LINC02936 and those co-transfected with sh-Scb or sh-SIX1#1, respectively (n = 3). (**D-F**) Relative lipid ROS (**D**), Fe^{2+} (**E**) and MDA (**F**) levels were measured in HEC1A cells stably transfected with mock, LINC02936 and those co-transfected with sh-Scb or sh-SIX1#1, respectively (n = 3).

(**G**) The SIX1 enrichment of CP in HEC1A cells stably transfected with mock, LINC02936 and those co-transfected with sh-Scb or sh-SIX1#1 were detected by ChIP assays (normalized to input), respectively (n = 3). (**H**) The mRNA levels of LINC02936, SIX1 and CP in HEC1A cells stably transfected with mock, LINC02936 and those co-transfected with sh-Scb or sh-SIX1#1 were detected by RT-qPCR, respectively (n = 3). (**I**) The protein levels of SIX1 and CP in HEC1A cells stably transfected with mock, LINC02936 and those co-transfected with sh-Scb or sh-SIX1#1 were detected by western blot, respectively (n = 3). Post-hoc Bonferroni's test after one-way ANOVA analysis was applied in panel **A-I**. * $P < 0.05$; ** $P < 0.01$; *** $P < 0.001$.

Table S1 Primer sets used for RT-qPCR, RIP and ChIP

Primer sets	Primers	Sequence (5'to 3')	Product size	Application
β-actin	Forward	AACTGGGACGACATGGAGAAAA	192	RT-qPCR
	Reverse	GGATAGCACAGCCTGGATAGCA		
LINC02936	Forward	AGTTTTTCTTCTCAGGGCCG	181	RT-qPCR
	Reverse	ACTGCCAGGAATAAAGCGAAGT		
SIX1	Forward	TGGTTTAAGAACCGGAGGCA	141	RT-qPCR
	Reverse	TTCTGAGCTGGACATGAGCG		
CP	Forward	CCAATACAAGCACAGGGGAGT	187	RT-qPCR
	Reverse	GCCAGATTTGGTGTCTTCATTT		
LINC02936	Forward	GCCTTTATGAGCTGTAACACTCACG	561	RT-PCR
	Reverse	TCATACTCAGGACTCTGTTTTTTTCA		
GAPDH	Forward	GTCAAGGCTGAGAACGGGAA	158	RT-qPCR
	Reverse	AAATGAGCCCCAGCCTTCTC		
U1	Forward	ACTTACCTGGCAGGGGAGATACC	137	RT-qPCR
	Reverse	CCACTACCACAAATTATGCAGTCG		
CP -1761/--1586	Forward	TCCTAGAAGGAGGCCTTGGG	176	ChIP
	Reverse	TACTTCAGCCCAGCTTCAGTTTCC		

Table S2 The foldchange and FDR value of six ferroptosis genes between different groups

Gene Symbol	Tumor vs. Normal		> 60 vs. ≤60		Dead vs. Alive	
	Log_2(FC)	FDR	Log_2(FC)	FDR	Log_2(FC)	FDR
CP	2.16	2.24E-07	0.84	8.45E-06	0.89	4.64E-04

SLC38A1	0.92	5.10E-05	0.46	1.04E-04	0.55	6.08E-04
CCDC6	0.73	9.10E-05	0.45	3.19E-06	0.46	6.74E-04
SIRT2	-0.45	2.94E-06	0.19	2.25E-03	0.26	3.00E-03
CIRBP	-1.15	2.58E-27	-0.20	4.87E-03	-0.30	6.79E-03
IL6	-1.53	7.24E-06	0.96	4.22E-05	1.45	3.26E-08

FC: Foldchange

Table S3 The foldchange and FDR value of eleven lncRNAs between different groups

Gene Symbol	Tumor vs. Normal		>60 vs. ≤60		G3 vs. G1-2		Dead vs. Alive	
	$Log_2(FC)$	FDR	$Log_2(FC)$	FDR	$Log_2(FC)$	FDR	$Log_2(FC)$	FDR
AC005256.1	2.57	7.08E-05	-1.07	1.47E-05	-0.89	6.22E-05	-1.26	8.70E-03
AC009237.15	1.04	4.58E-03	0.95	1.66E-08	1.05	1.39E-11	0.93	5.52E-06
AP003306.2	1.51	1.48E-04	-0.59	1.33E-03	-1.20	1.00E-15	-1.04	2.87E-04
FP671120.6	3.53	2.06E-03	-4.81	1.34E-36	3.21	5.01E-11	-3.74	2.07E-06
KC877982.1	3.49	2.84E-03	1.60	1.72E-03	1.99	9.58E-06	-3.86	1.21E-06
LCAL1	3.17	3.29E-06	0.81	3.77E-03	1.18	1.49E-06	1.65	2.97E-09
LINC00942	2.91	1.07E-04	-0.89	3.46E-03	2.46	4.68E-19	1.66	4.23E-06
LINC01224	2.61	3.32E-09	0.62	4.49E-04	1.36	4.28E-19	0.65	6.53E-03
LINC02381	-1.01	3.26E-03	0.94	1.95E-05	1.67	3.15E-18	0.87	2.66E-03
LINC02936	1.50	2.44E-04	1.05	7.96E-06	1.62	1.73E-15	1.82	6.20E-06
PRRT3-AS1	0.86	6.79E-03	0.39	8.79E-03	0.81	7.72E-11	0.71	7.35E-05

FC: Foldchange

Table S4 The foldchange and FDR value of eight TFs between different groups

Gene Symbol	Tumor vs. Normal		>60 vs. ≤60		G3 vs. G1-2		Dead vs. Alive	
	$Log_2(FC)$	FDR	$Log_2(FC)$	FDR	$Log_2(FC)$	FDR	$Log_2(FC)$	FDR
CEBPG	0.38	9.62E-03	0.38	2.85E-07	0.59	1.87E-19	0.31	8.50E-03
GLIS2	-0.68	3.37E-04	0.34	6.42E-03	0.94	6.31E-21	0.59	2.83E-04
IKZF3	1.39	1.65E-04	0.83	1.41E-06	0.88	1.20E-08	1.26	4.95E-11
MEOX1	-1.44	2.08E-05	1.30	2.05E-08	2.25	2.45E-27	1.21	3.30E-05
MESP2	2.76	1.64E-07	1.17	8.95E-09	0.56	3.36E-03	1.25	3.06E-07
SIX1	3.59	2.42E-13	0.38	8.28E-03	0.86	3.29E-07	0.82	1.39E-03
SIX3	3.45	5.89E-07	2.03	2.62E-13	1.95	2.98E-14	1.17	2.63E-03
ZNF516	-0.97	6.84E-07	-0.54	1.14E-05	-0.89	6.06E-18	-0.67	7.02E-04

FC: Foldchange

Table S5 Protein sequence score based on PPRInt and hybridNAP program

No.	Sequence	PPRInt	hybridNAP			
		SVM value	Pred	RAA	RSA	ECO
1	G	-0.6033	0.310	0.094	0.964	0.889
2	E	-0.5948	0.143	0.045	0.635	0.883
3	E	-0.7612	-0.092	0.052	0.531	0.399
4	T	-0.0978	-0.053	0.117	0.366	0.656

5	S	-0.0474	-0.134	0.225	0.347	0.404
6	Y	0.2346	0.100	0.345	0.355	0.920
7	C	-0.2923	-0.112	0.360	0.224	0.521
8	F	0.01749	0.004	0.398	0.296	0.709
9	K	-0.1742	-0.081	0.347	0.429	0.365
10	E	-0.3734	-0.134	0.240	0.475	0.241
11	K	0.33798	-0.142	0.263	0.493	0.184
12	S	-0.7333	-0.121	0.417	0.299	0.373
13	R	-0.5119	-0.017	0.562	0.468	0.348
14	G	-0.5325	-0.197	0.380	0.321	0.175
15	V	-1.3321	-0.249	0.200	0.276	0.207
16	L	-0.3645	-0.000	0.410	0.168	0.844
17	R	-0.7001	-0.008	0.555	0.439	0.409
18	E	-1.1694	-0.129	0.395	0.443	0.195
19	W	-1.0426	-0.009	0.413	0.216	0.762
20	Y	-1.2781	-0.027	0.423	0.269	0.646
21	A	-1.0838	-0.206	0.302	0.450	0.046
22	H	-0.388	-0.086	0.320	0.458	0.335
23	N	-0.3435	-0.095	0.253	0.338	0.495
24	P	0.8087	0.061	0.253	0.518	0.682
25	Y	0.85029	0.121	0.340	0.449	0.865
26	P	0.07587	0.093	0.235	0.501	0.795
27	S	0.74217	-0.063	0.130	0.426	0.550
28	P	0.91078	-0.044	0.355	0.493	0.379
29	R	0.44052	-0.028	0.542	0.537	0.249
30	E	-0.0822	-0.031	0.372	0.401	0.510
31	K	0.28895	0.028	0.487	0.362	0.637
32	R	0.13149	-0.059	0.620	0.449	0.226
33	E	-0.7963	-0.178	0.307	0.408	0.165
34	L	-1.1978	-0.127	0.130	0.234	0.616
35	A	-0.9611	-0.163	0.100	0.265	0.504
36	E	-0.6569	-0.145	0.050	0.578	0.209
37	A	-0.8278	-0.226	0.092	0.453	0.123
38	T	-0.2661	-0.029	0.150	0.453	0.594
39	G	-0.7533	0.059	0.172	0.597	0.633
40	L	-0.6622	-0.007	0.188	0.353	0.745
41	T	-0.7018	-0.043	0.188	0.468	0.515
42	T	-0.7882	-0.212	0.180	0.419	0.145
43	T	-0.6839	-0.096	0.180	0.570	0.263
44	Q	-0.1086	0.172	0.190	0.605	0.904
45	V	-0.5298	0.167	0.204	0.799	0.651

The green background values for RAA, RSA and ECO indicate a high probability for binding, while the red background values indicate the opposite based on hybridNAP program.

Table S6 SIX1 and CP expression score in human EC tissues

Grorp	N=48	SIX1		χ2	P	CP		χ2	P
		−～+	+ +～+ ++			−～+	+ +～+++		
Age									
≤60	23	12	11	4.057	0.044	14	9	5.259	0.022
＞60	25	6	19			7	18		
Grade									
G1-2	29	16	13	9.763	0.002	17	12	6.583	0.010
G3	19	2	17			4	15		
Stage									
1-2	34	14	20	0.672	0.412	12	22	3.387	0.066
3-4	14	4	10			9	5		
Histological subtypes									
Endometrioid carcinoma	32	13	19	0.400	0.527	18	14	6.095	0.014
Non-endometrioid carcinoma	16	5	11			3	13		
Lymph node metastasis									
No	37	12	25	1.769	0.288*	15	22	0.676	0.498*
Yes	11	6	5			6	5		

*: Comparisons were performed using Fisher's exact test.
